# A first order method for linear programming parameterized by circuit imbalance

**DOI:** 10.1007/s10107-025-02264-7

**Published:** 2025-08-19

**Authors:** Richard Cole, Christoph Hertrich, Yixin Tao, László A. Végh

**Affiliations:** 1 Courant Institute, New York University, NY, 10012 USA; 2 University of Technology Nuremberg, 90461 Nürnberg, Germany; 3 ITCS, Key Laboratory of Interdisciplinary Research of Computation and Economics, Shanghai University of Finance and Economics, Shanghai, 200433 China; 4 Hertz Chair in Algorithms and Optimization, University of Bonn, 53121 Bonn, Germany; 5 London School of Economics and Political Science, London WC2A 2AE, UK; 6 Corvinus Institute for Advanced Studies, Corvinus University, Budapest, 1093 Hungary, Hungary

**Keywords:** Linear Programming, First Order Methods, Hoffman Proximity, Circuit Imbalances, 90C05, 90C25, 90C06, 65K05, 52B05

## Abstract

Various first order approaches have been proposed in the literature to solve Linear Programming (LP) problems, recently leading to practically efficient solvers for large-scale LPs. From a theoretical perspective, linear convergence rates have been established for first order LP algorithms, despite the fact that the underlying formulations are not strongly convex. However, the convergence rate typically depends on the Hoffman constant of a large matrix that contains the constraint matrix, as well as the right hand side, cost, and capacity vectors. We introduce a first order approach for LP optimization with a convergence rate depending polynomially on the circuit imbalance measure, which is a geometric parameter of the constraint matrix, and depending logarithmically on the right hand side, capacity, and cost vectors. This provides much stronger convergence guarantees. For example, if the constraint matrix is totally unimodular, we obtain polynomial-time algorithms, whereas the convergence guarantees for approaches based on primal-dual formulations may have arbitrarily slow convergence rates for this class. Our approach is based on a fast gradient method due to Necoara, Nesterov, and Glineur (Math. Prog. 2019); this algorithm is called repeatedly in a framework that gradually fixes variables to the boundary. This technique is based on a new approximate version of Tardos’s method, that was used to obtain a strongly polynomial algorithm for combinatorial LPs (Oper. Res. 1986).

## Introduction

In this paper, we develop new first order algorithms for approximately solving the linear program 
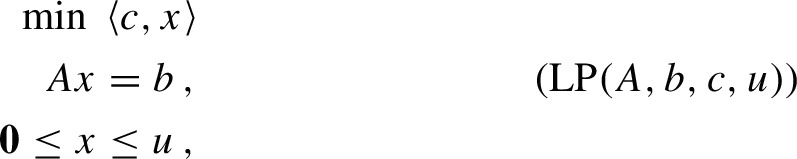
 where $A\in {\mathbb {R}}^{m\times n}$, $b\in {\mathbb {R}}^m$, $c,u\in {\mathbb {R}}^n$. We assume that $m\le n$. We use the notation $[{\textbf{0}},u]=\{x\in {\mathbb {R}}^n\mid {\textbf{0}}\le x\le u\}$, and denote the feasible region as ${\mathcal {P}}_{A,b,u}{:}{=}\{x\in {\mathbb {R}}^n\mid \, Ax=b\, ,\, x\in [{\textbf{0}},u]\}$.

Linear programming (LP) is one of the most fundamental optimization problems with an immense range of applications in applied mathematics, operations research, computer science, and more. While Dantzig’s Simplex method works well in practice, its running time may be exponential in the worst case. Breakthrough results in the 1970s and 1980s led to the development of the first polynomial time algorithms, the ellipsoid method [14] and interior point methods (IPMs) [13]. The Simplex method was one of the earliest computations implemented on a computer, and there are highly efficient LP solvers available, based on Simplex and interior point methods.

Linear programming can also be seen as a special case of more general optimization models: it can be captured by various convex programs, saddle point problems, and linear complementarity problems. Due to these connections, the development of new LP algorithms has been an important driving force in the development of optimization theory.

In this paper, we focus on first order methods (FOMs) for LP. The benefit of FOMs is cheap iteration complexity and efficient implementability for large-scale problems. In contrast to IPMs, they do not require careful initialization. FOMs are prevalent in optimization and machine learning, but they are not an obvious choice for LP for two reasons. First, the standard formulation has a complicated polyhedral feasible region, and therefore standard techniques are not directly applicable. Second, FOMs usually do not lead to polynomial running time guarantees: this is in contrast with IPMs that are polynomial and also efficient in practice.

Nevertheless, FOMs turn out to be practically efficient for large-scale LPs. In a recent paper Applegate et al. [1] use a restarted primal-dual hybrid gradient (PDHG) method based on a saddle point formulation. Their implementation outperforms the state-of-the art commercial Simplex and IPM solvers on standard benchmark instances, and is able to find high accuracy solutions to large-scale PageRank instances.

The number of iterations needed to find an $\varepsilon$-approximate solution in standard FOMs is typically $O(1/\varepsilon )$ or $O(1/\sqrt{\varepsilon })$. However, strong convexity properties can yield *linear convergence*, i.e., an $O(\log (1/\sqrt{\varepsilon }))$ dependence. No strongly convex formulation is known to capture LP. Despite this, there is a long line of work on FOMs that achieve linear convergence guarantees for LP, starting from Eckstein and Bertsekas’s alternating direction method from 1990 [6], followed by a variety of other techniques, e.g., [1, 2, 10, 11, 15, 22, 23].

Before discussing these approaches, let us specify the notion of approximate solutions. By a $\delta$-*feasible* solution, we mean an $x\in [{\textbf{0}},u]$ with $\Vert Ax-b\Vert _1\le \delta \Vert A\Vert _1$. If LP(*A*, *b*, *c*, *u*) is feasible, we let $\Phi (A,b,c,u)$ denote the optimum value. A $\delta$-*optimal* solution satisfies $\left\langle c,x\right\rangle \le \Phi (A,b,c,u)+\delta \Vert c\Vert _\infty$. Our goal will be to find a $\delta$-feasible and $\delta$-optimal solution for a required accuracy $\delta >0$.^1^

The above mentioned works are able to find $\delta$-feasible and $\delta$-optimal solutions in running times that depend polynomially on $\log (1/\delta )$, *n*, and *C*(*A*, *b*, *c*, *u*), a constant depending on the problem input. In particular, Applegate, Hinder, Lu, and Lubin [2] give a running time bound $O(C\log (1/\delta ))$ for restarted PDHG, where *C* is the Hoffman-constant associated with the primal-dual embedding of the LP. Recently, for the case when *A* is a totally unimodular matrix and there are no upper bounds *u*, Hinder [11] bounded the running time of restarted PDHG as $O(H n^{2.5}\sqrt{\textrm{nzz}(A)}\log (Hm/\delta ))$, where $\textrm{nzz}(A)$ is the number of nonzero entries of *A*, and *b*, *c* are integer vectors with $\Vert b\Vert _\infty ,\Vert c\Vert _\infty \le H$.

However, the constants involved in the running time bound are typically not polynomial in the binary encoding length of the input. In this paper, we give the first FOM-based algorithm with polynomial dependence on $\log (1/\delta )$, *n*, a constant ${\bar{\kappa }}({\mathcal {X}}_A)$, and $\log \Vert b\Vert$, $\log \Vert c\Vert$, and $\log \Vert u\Vert$, as stated in Theorem 1.1 below. The constant ${\bar{\kappa }}({\mathcal {X}}_A)$ is the *max circuit imbalance measure* defined and discussed below. In particular, it is upper bounded by the maximal subdeterminant $\Delta (A)$, but it is often much smaller than $\Delta (A)$. For totally unimodular matrices, we have ${\bar{\kappa }}({\mathcal {X}}_A)=1$. Note that the running time depends polynomially on the logarithms of the capacity and cost vectors. In contrast, the bound in [11] only applies for the totally unimodular case and the running time is linear in $\Vert b\Vert _\infty +\Vert c\Vert _\infty$.

For technical convenience, we will assume throughout that $\Vert A\Vert _1\ge 1$. In general, our results still hold by replacing $\Vert A\Vert _1$ with $\max \{\Vert A\Vert _1, 1\}$ in the statement of the following theorem.^2^ Note that since the system LP(*A*, *b*, *c*, *u*) is bounded, whenever feasible, it has an optimal solution.

### Theorem 1.1

Consider an instance of  LP(*A*, *b*, *c*, *u*) with $\Vert A\Vert _1\ge 1$. There is an FOM-based algorithm that obtains a solution *x* that is $\delta$-feasible and $\delta$-optimal, or concludes that no feasible solution exists, and whose runtime is dominated by the cost of performing $O\big ( n^{1.5} m^2 \Vert A\Vert _1^2 \cdot {\bar{\kappa }}^3({\mathcal {X}}_{A})$ $\log ^3 \left( (\Vert u\Vert _1+\Vert b\Vert _1) n m \cdot \kappa ({\mathcal {X}}_A) \Vert A\Vert _1 /\delta \right) \big )$ gradient descent updates. Additionally, our algorithm returns a dual solution certifying approximate optimality of the solution in $O\big (m \Vert A\Vert _2 \cdot {\bar{\kappa }}({\mathcal {X}}_A)\cdot \log (n \Vert c\Vert _1 / \delta )\big )$ gradient descent updates.

***Hoffman bounds and quadratic function growth.***

The main underlying tool for proving linear convergence bounds is Hoffman-proximity theory, introduced by Hoffman in 1952 [12]. Let $A\in {\mathbb {R}}^{m\times n}$, let $\Vert .\Vert _\alpha$ be a norm in ${\mathbb {R}}^m$ and $\Vert .\Vert _\beta$ be a norm in ${\mathbb {R}}^n$. Then there exists a constant $\theta _{\alpha ,\beta }(A)$ such that for any $x\in [{\textbf{0}},u]$, and any $b\in {\mathbb {R}}^m$, whenever ${\mathcal {P}}_{A,b,u}$ is nonempty, there exists an ${{\bar{x}}}\in {\mathcal {P}}_{A,b,u}$ such that

$$\begin{aligned} \Vert {{\bar{x}}}-x\Vert _\beta \le \theta _{\alpha ,\beta }(A)\Vert Ax-b\Vert _\alpha \, . \end{aligned}$$

To see how such bounds can lead to linear convergence, let us first focus on finding a feasible solution in ${\mathcal {P}}_{A,b,u}$. This can be formulated as a convex quadratic minimization problem:

1 $$\begin{aligned} \begin{aligned} \min \tfrac{1}{2}\Vert Ax-b\Vert ^2\quad \text{ s.t. }\quad x\in [{\textbf{0}},u]\, . \end{aligned} \end{aligned}$$

This is a smooth objective function, but not strongly convex. Nevertheless, Hoffman-proximity guarantees that for any $x\in [{\textbf{0}},u]$ where $f(x){:}{=}\frac{1}{2}\Vert Ax-b\Vert ^2$ is close to the optimum value, there exists *some* optimal solution ${{\bar{x}}}$ nearby. Necoara, Nesterov, and Glineur [15] introduce various relaxations of strong convexity, including the notion of $\mu _f$-quadratic growth (Definition 2.3), and show that these weaker properties suffice for linear convergence.

Hence, [15] implies that a $\delta$-feasible LP solution can be found by a Fast Gradient Method with Restart (R-FGM) in $O(\Vert A\Vert _2 \cdot \theta _{2,2}(A)\log (m\Vert b\Vert _1/\delta ))$ iterations. If *A* is a totally unimodular (TU) matrix, the dependence is given by $\theta _{2,2}(A)\le m$ (see Lemma 3.6).

To solve  LP(*A*, *b*, *c*, *u*), [15] in effect uses the standard reduction from optimization to feasibility by writing the primal and dual systems together. By strong duality, if LP(*A*, *b*, *c*, *u*) is feasible and bounded, then *x* is a primal and $(\pi ,w^+,w^-)$ is a dual optimal solution if and only if

2 $$\begin{aligned} Ax=b\, ,\quad A^\top \pi + w^--w^+=c\, , \quad \left\langle c,x\right\rangle -\left\langle b,\pi \right\rangle +\left\langle u,w^+\right\rangle =0\, ,\quad x,w^-,w^+\ge 0\,. \end{aligned}$$

We can use R-FGM for this larger feasibility problem. However, the constraint matrix *M* now also includes the vectors *c*, *b*, and *u*. In particular, while $\theta _{2,2}(A)$ is small for a TU matrix, $\theta _{2,2}(M)$ may be unbounded, as shown in Sect. 10.

Other previous works obtain linear convergence bounds using different approaches, but share the above characteristics: their running time includes a constant term *C*(*A*, *b*, *c*, *u*). For example, [6] and [22] use an alternating direction method based on an augmented Lagrangian, and [2] and [11] use restart PDHG. The convergence bounds depend not only on the Hoffman-constant of the system, but also linearly on the maximum possible norm of the primal and dual iterates seen during the algorithm.

### Our approach

We present an algorithm in the FOM family with polynomial dependence on $\log (1/\delta )$, *n*, *m*, $\log \Vert u\Vert$, $\log \Vert b\Vert$, $\log \Vert c\Vert$ and a constant *C*(*A*) only dependent on *A*. Our algorithm repeatedly calls R-FGM, described in [15], on a potential function of the form

3 $$\begin{aligned} F_{\tau }(x) {:}{=} \frac{1}{2}(\max \{0, \langle {\hat{c}}, x \rangle - \tau \})^2 + \frac{1}{2\Vert A\Vert _1^2} \Vert Ax - b\Vert _2^2\, , \end{aligned}$$

for a suitably chosen parameter $\tau \in {\mathbb {R}}$, and a modified cost function ${\hat{c}}$. If we use ${\hat{c}}=c/\Vert c\Vert _\infty$, and $\tau$ is slightly below the optimum value, then one can show that a near-minimizer *x* of $F_{\tau }(x)$ is a near optimal primal solution to the original LP, and moreover, we can extract a near-optimal dual solution from the gradient $\nabla F_{\tau }(x)$.

Thus, one could find a $\delta$-approximate and $\delta$-optimal solution to LP(*A*, *b*, *c*, *u*) with $\log (1/\delta )$ dependence by doing a binary search over the possible values of $\tau$, and running R-FGM for each guess. This already improves on the parameter dependence, however, it still involves a constant *C*(*A*, *c*). This is because the minimization of $F_{\tau }(x)$ can be casted in the form (1) with the matrix $$\begin{pmatrix}A &  0\\ \Vert A\Vert _1 c^\top &  \Vert A\Vert _1\end{pmatrix}$$. The resulting Hoffman constant can be arbitrarily worse than the one for the original system.

To overcome this issue, we instead define ${\hat{c}}$ as an $\varepsilon$-discretization of $c/\Vert c\Vert _\infty$. We show that the Hoffman constant remains bounded in terms of the Hoffman constant of the feasibility system and a suitably chosen $\varepsilon >0$. Now, for the appropriate choice of $\tau$, a near-minimizer of $F_{\tau }(x)$ only gives a crude approximation to the original LP: the error depends on the discretization parameter $\varepsilon$, and to keep the Hoffman constant under control we cannot choose $\varepsilon$ very small. Nonetheless, the dual solution obtained from the gradient contains valuable information. For certain indices $i\in N$, using primal-dual slackness, one can conclude $x^*_i\approx 0$ or $x_i^*\approx u_i$ for an optimal solution $x^*$ to the original LP. We fix all such $x_i$ to 0 or $u_i$, respectively, and recurse. Even if we not find any such $x_i$, we make progress by replacing our cost function by an equivalent reduced cost with the $\ell _\infty$ norm decreasing by at least a factor two.

To summarize: our overall algorithm has an outer loop that gradually fixes the variables to the upper and lower bounds, and repeatedly replaces the cost by a reduced cost. In the inner loop, we call R-FGM in a binary search framework that guesses the parameter $\tau$. We note that while R-FGM is run on a number of systems, the total number of these systems is logarithmically bounded in $1/\delta$ and the input parameters. Moreover, besides the first order updates, we only perform simple arithmetic operations: based on the gradient, we eliminate a subset of variables and shift the cost function. On a high level, our algorithm is a repeatedly applied FOM, where after each run, we ‘zoom in’ to a ‘critical’ part of the problem based on what we learned from the previous iteration.

***Circuit imbalance measures and proximity***

The key condition numbers for our algorithm are *circuit imbalance measures*. For a linear space $W\subseteq {\mathbb {R}}^n$, an elementary vector is a support minimal nonzero vector in *W*. The *(fractional) circuit imbalance measure* $\kappa (W)$ is the largest ratio between the absolute values of two entries of an elementary vector. If *W* is a rational space, then every elementary vector can be rescaled to have integer entries; and the *max circuit imbalance measure* ${\bar{\kappa }}(W)$ is the smallest integer *k* such that all elementary vectors can be scaled to have integer entries between $-k$ and *k*. Note that $\kappa (W)\le {\bar{\kappa }}(W)$. For a matrix *A*, we also use $\kappa (A)=\kappa (\ker (A))$ and ${\bar{\kappa }}(A)={\bar{\kappa }}(\ker (A))$. We give a more detailed introduction to these measures in Sect. 3. We define the subspace ${\mathcal {X}}_A=\ker (A|-I_m)$; thus, $(v,-Av)\in {\mathcal {X}}_A$ for any $v\in {\mathbb {R}}^n$.

Circuit imbalances play two roles in our algorithm. First, they are used to bound the number of iterations of R-FGM. The circuit imbalance measure of ${\mathcal {X}}_A$ gives the Hoffman-proximity bound $\theta _{1,\infty }(A)\le \kappa ({\mathcal {X}}_A)$ (see Lemma 3.6). To bound the number of iterations in R-FGM, we need a Hoffman bound—equivalently, a circuit imbalance bound—for the matrix $$B=\begin{pmatrix}A &  0\\ \Vert A\Vert _1 {\hat{c}}^\top &  \Vert A\Vert _1\end{pmatrix}$$, where ${\hat{c}}$ is the $\varepsilon$-discretization of $c/\Vert c\Vert _\infty$. $\kappa ({\mathcal {X}}_B)$ can be bounded in terms of the max circuit imbalance measure ${\bar{\kappa }}({\mathcal {X}}_A)$, namely, $\kappa ({\mathcal {X}}_B)\le 2m\cdot {\bar{\kappa }}^2({\mathcal {X}}_A)/\varepsilon$.

The second role of $\kappa ({\mathcal {X}}_A)$ is for the variable fixing argument in the outer loop of the algorithm. Recall that the inner loop returns a near optimal primal solution with respect to the rounded cost, as well as a near optimal dual solution derived from the gradient of the potential function. We would like to infer that variables with a large positive or negative dual slack can be rounded to the lower or upper bounds. To make such an inference, the rounding accuracy $\varepsilon$ needs to be calibrated to $\kappa ({\mathcal {X}}_A)$. The larger $\kappa ({\mathcal {X}}_A)$ is, the more refined the rounding is needed to obtain such guarantees.

***Guessing the condition numbers.***

Our algorithm requires explicit bounds on the circuit imbalance measures both in the inner and outer loops. However, the circuit imbalance measures cannot be approximated even within an exponential factor unless $P=NP$ (see Sect. 3.1). Similar issues arise in several algorithms that rely on condition numbers. In particular, R-FGM in [15] explicitly requires a bound on the Hoffman constant to determine the step-length; it does not address how such a bound could be obtained.^3^

We circumvent this problem by a standard doubling guessing procedure. We first run the algorithm with the initial guess $\hat{\kappa }=1$. Either it succeeds, or otherwise we restart after doubling the guess ${\hat{\kappa }}$; and so forth, until the algorithm first succeeds. The asymptotic running time is the same as when the circuit imbalance value is known. The only nontrivial issue is checking whether we succeeded; this can be done by running a final dual feasibility algorithm.

We note that the algorithm may succeed even if ${\hat{\kappa }}$ is much better than the actual ${\bar{\kappa }}({\mathcal {X}}_A)$ value. In fact, the guessing procedure is a natural heuristic. We initially start with a crude discretization strategy to guess variable fixings from the outputs of R-FGM. In the event this leads to an infeasible or suboptimal solution, we restart after increasing the accuracy.

### Related work

We recall that an LP algorithm is strongly polynomial if it only uses basic arithmetic operations ($+,-,\times ,/$) and comparisons, and the number of such operations is polynomial in the number of variables and constraints. Further, the algorithm must be in PSPACE, that is, the size of the numbers appearing in the computation must remain bounded in the input size. The existence of a strongly polynomial algorithm for LP is on Smale’s list of main challenges for 21st century mathematics [17].

The variable fixing idea in our algorithm traces its roots to Tardos’s strongly polynomial algorithm for minimum-cost circulations. The same idea was extended by Tardos [19] to obtain a poly$(n,\log \Delta (A))$ time algorithm for finding an exact solution to LP(*A*, *b*, *c*, *u*) for an integer constraint matrix $A\in {\mathbb {Z}}^{m\times n}$ with largest subdeterminant $\Delta (A)$. This running time bound is strongly polynomial for ‘combinatorial LPs’, that is, LPs with all entries being integers of absolute value poly(*n*).

We note that $\kappa (A)\le \kappa ({\mathcal {X}}_A)\le {\bar{\kappa }}({\mathcal {X}}_A)\le \Delta (A)$ for an integer matrix *A*. Dadush et al. [4] strengthened Tardos’ result by replacing $\Delta (A)$ by $\kappa (A)$, and removing all integrality-based arguments, and obtained a poly$(n,\log \kappa (A))$ running time bound. The algorithm is of black-box nature, and can use any LP solver; an exact optimal solution can be found by running *nm* LP-solvers to accuracy $\delta =1/\textrm{poly}(n,\log \kappa (A))$.

Our algorithm uses variable fixing in a different manner, giving a robust extension to the approximate setting. Our end goal is not an exact optimal solution, but rather an approximate one. The approximate solution obtained from the FOM in the inner loop has weaker guarantees. Tardos [19] also uses subproblems with a similarly rounded cost function, but requires exact feasibility, which cannot be obtained from an FOM.

For this reason, we obtain weaker guarantees, e.g., we may fix variables to 0 that are small but positive in all optimal solutions. However, this is acceptable if we are only aiming for an approximate solution. On the positive side, we only need a logarithmic number of executions of the outer loop, in contrast to *nm* in [4, 19]. This is because for us it is already sufficient progress to decrease the norm of the reduced cost, even if we cannot fix any variables.

A poly$(n,\log \kappa (A))$ running time for LP can also be achieved by a special class of ‘combinatorial’ interior point methods, called *Layered Least Squares (LLS) IPMs*. This class was introduced by Vavasis and Ye [21]. The parameter dependence was on the Dikin–Stewart–Todd condition measure ${\bar{\chi }}(A)$, but [5] observed that the two condition numbers are close to each other. Further, they gave a stronger LLS IPM with running time dependent on the optimal value $\kappa ^*(A)$ of $\kappa (A)$ achievable by column rescaling. We refer the reader to the survey [7] for further results related to circuit imbalances and their uses in LP, including also diameter and circuit diameter bounds.

We also note that Fujishige et al. [8] recently gave a poly$(n,\kappa (A))$ algorithm for the minimum norm point problem (1) by combining FOMs and active set methods. Their algorithm terminates with an exact solution; on the other hand, it also uses projection steps that involve solving a system of linear equations. Thus, it is not an FOM; moreover, it is not applicable for optimization LP.

***Notation.***

We let $[n]=\{1,2,\ldots ,n\}$. For a vector $x\in {\mathbb {R}}^n$, let $\textrm{supp}(x)=\{i\in [n]\mid x_i\ne 0\}$ denote its support. For $A\in {\mathbb {R}}^{m\times n}$, let $A_i$ denote the *i*-th column of *A*. We use the norm $\Vert A\Vert _1=\max _{j\in [n]}\sum _{i=1}^m |a_{ij}|$, and the spectral norm $\Vert A\Vert _2$. We normalize the input matrix so that $\Vert A\Vert _1\ge 1$. For a linear subspace $W\subseteq {\mathbb {R}}^n$, we let $W^\perp \subseteq {\mathbb {R}}^n$ denote the orthogonal complement.

***Overview.***

The remainder of the paper is structured as follows. Sect. 2 is on preliminaries regarding the convergence guarantees of R-FGM [15]. Sect. 3 discusses further preliminaries regarding circuit imbalances, proximity and their algorithmic uses. Sect. 4 is a more detailed overview of our main ideas including formal statements; the algorithm is formally presented Sect. 5, where the main result Theorem 1.1 is also proved. Sect. 6 contains proofs related to the crucial proximity results of Sect. 4. The main statements for the outer and inner routines are proved in Sects. 7 and 8, respectively. The proofs related to dual certification are in Sect. 9. Finally, Sect. 10 provides an example showing that a simple self-dual embedding can blow up the Hoffman constant, serving as a motivation for our work.

## Linear convergence for functions with quadratic growth

Assume we are interested in finding a $\delta$-feasible solution to LP(*A*, *b*, *c*, *u*) or concluding that the system is infeasible. We consider the convex formulation

4 $$\begin{aligned} \begin{aligned} \min \tfrac{1}{2}\Vert Ax-b\Vert ^2_2\\ {\textbf{0}}\le x\le u\, . \end{aligned} \end{aligned}$$

We now outline the running time bounds obtained by Neocara, Nesterov, and Glineur [15].

### Definition 2.1

The function $f:\, {\mathbb {R}}^n\rightarrow {\mathbb {R}}$ is $L_f$-*smooth* or has $L_f$-*Lipschitz continuous gradient* if $\Vert \nabla f(x) - \nabla f(y)\Vert _2 \le L_f \cdot \Vert x - y\Vert _2$ for any $x,y\in \operatorname {dom}(f)$.

### Lemma 2.2

The function $f(x) = \frac{1}{2} \Vert A x - b\Vert _2^2$ is $\Vert A\Vert _2^2$-smooth.

### Proof

This follows as $\Vert \nabla f(x) - \nabla f(y)\Vert _2 = \Vert A^\top A (x - y)\Vert _2 \le \Vert A^\top A\Vert _2 \cdot \Vert x - y\Vert _2=\Vert A\Vert _2^2\cdot \Vert x - y\Vert _2$. $\square$

### Definition 2.3

Let $f:{\mathbb {R}}^n\rightarrow {\mathbb {R}}$ be continuously differentiable, let $X\subseteq \operatorname {dom}(f)$ be a closed convex set, and $f^*=\min _{x\in X} f(x)$ the minimum value. Then *f* has $\mu _f$-*quadratic growth on* *X* if, for any $x\in X$, there exists an optimal solution ${\bar{x}}$ (that is, $f({\bar{x}}) = f^*$) such that $f(x) - f^* \ge \frac{\mu _f}{2} \Vert x - {\bar{x}}\Vert _2^2$.

### Lemma 2.4

( [15, Theorem 8]) The function $f(x) = \frac{1}{2} \Vert A x - b\Vert _2^2$ has $1 / \theta ^2_{2,2}(A)$-quadratic growth.

The R-FGM method proposed in [15] optimizes a convex function by iteratively applying the standard accelerated projected gradient descent algorithm. R-FGM starts with $x^0$ as the initial point and then performs $h_{\scriptscriptstyle {\!R}}$ iterations of accelerated projected gradient descent to obtain $x^{h_{\scriptscriptstyle {\!R}}}$, for a suitable $h_{\scriptscriptstyle {\!R}}$. R-FGM then uses $x^{h_{\scriptscriptstyle {\!R}}}$ as the new starting point and repeats the process, performing another $h_{\scriptscriptstyle {\!R}}$ iterations of accelerated projected gradient descent. This process is repeated multiple times. For a convex function which is $L_f$-smooth and has $\mu _f$-quadratic growth, [15] shows that, for every $h_{\scriptscriptstyle {\!R}}=O(\sqrt{L_f/\mu _f})$ iterations, the difference between the current function value and the optimum is reduced by a factor of $\textrm{e}^2$:

### Theorem 2.5

Suppose function *f* is $L_f$-smooth and has $\mu _f$-quadratic growth. Let $h_{\scriptscriptstyle {\!R}}= \lceil 2 \textrm{e}\sqrt{L_f / \mu _f} \rceil$ and $x^0$ is the starting point. Then, after $k \cdot h_{\scriptscriptstyle {\!R}}$ iterations, the R-FGM method outputs *x* such that

$$\begin{aligned} f(x) - f^* \le \textrm{e}^{-2 k} (f(x^0) - f^*). \end{aligned}$$

## Circuit imbalances and proximity

For a linear space $W\subset {\mathbb {R}}^n$, $g\in W$ is an *elementary vector* if *g* is a support minimal nonzero vector in *W*, that is, no $h\in W\setminus \{{\textbf{0}}\}$ exists such that $\textrm{supp}(h)\subsetneq \textrm{supp}(g)$. A *circuit* in *W* is the support of some elementary vector. We let ${\mathcal {F}}(W)\subseteq W$ denote the set of elementary vectors in the space *W*.^4^

The subspaces $W=\{{\textbf{0}}\}$ and $W={\mathbb {R}}^N$ are called trivial subspaces; all other subspaces are nontrivial. We define the *fractional circuit imbalance measure*

$$\begin{aligned} \kappa (W){:}{=}\max \left\{ \left| \frac{g_j}{g_i}\right| :\, g\in {\mathcal {F}}(W), i,j\in \textrm{supp}(g)\right\} \, \end{aligned}$$

for nontrivial subspaces, and $\kappa (W){:}{=}1$ for trivial subspaces.

Further, if *W* is a rational linear space, we let ${\bar{{\mathcal {F}}}}(W)\subseteq {\mathcal {F}}(W)$ denote the set of integer elementary vectors $g\in {\mathbb {Z}}^n\cap {\mathcal {F}}(W)$ such that the largest common divisor of the entries is 1. We define the *max circuit imbalance measure* as

$$\begin{aligned} {\bar{\kappa }}(W){:}{=}\max \left\{ \Vert g\Vert _\infty :\, g\in {\bar{{\mathcal {F}}}}(W)\right\} \, . \end{aligned}$$

When using the term ‘circuit imbalance measure’ without any specification, it will refer to the fractional version. Note that $\kappa (W)\le {\bar{\kappa }}(W)$ but they may not be equal. For example, if the single elementary vector up to scaling is (4, 7, 8), then $\kappa (W)=2$ but ${\bar{\kappa }}(W)=8$.

Let $A\in {\mathbb {R}}^{m\times n}$ be a matrix, and let $W=\ker (A)$ be the kernel space of *A*. We let ${\mathcal {F}}(A)$, $\kappa (A)$, ${\bar{\kappa }}(A)$ denote ${\mathcal {F}}(W)$, $\kappa (W)$, ${\bar{\kappa }}(W)$, respectively, for the kernel space $W=\ker (A)$. We refer the reader to the survey [7] for properties and applications of circuit imbalances. Below, we mention some basic properties.

Recall that a matrix is *totally unimodular (TU)* if the determinant of every square submatrix is 0, $+1$, or $-1$. We note that $\kappa (W)={\bar{\kappa }}(W) = 1$ if and only if there exists a TU matrix $A\in {\mathbb {R}}^{m\times n}$ such that $W = \ker (A)$. This follows by a 1957 result of Cederbaum. Further, it is easy to verify that for an integer matrix $A\in {\mathbb {Z}}^{m\times n}$, the inequality ${\bar{\kappa }}(A)\le \Delta (A)$ holds, where $\Delta (A)$ is the largest absolute value of a subdeterminant of *A*. However, ${\bar{\kappa }}(A)$ can be arbitrarily smaller: ${\bar{\kappa }}(A)=2$ for the node-edge incidence matrix of any undirected graph, whereas $\Delta (A)$ can be exponentially large. See [7, Section 3.1] for the above results. We will also use the following important self-duality of $\kappa$:

### Lemma 3.1

( [5]) Let $W\subseteq {\mathbb {R}}$ be a linear subspace. Then $\kappa (W)=\kappa (W^\perp )$.

***Conformal circuit decompositions***

We say that the vector $y \in {\mathbb {R}}^n$ *conforms to* $x\in {\mathbb {R}}^n$ if $x_i y_i > 0$ whenever $y_i\ne 0$. Given a subspace $W\subseteq {\mathbb {R}}^n$, a *conformal circuit decomposition* of a vector $z\in W$ is a decomposition $z=\sum _{k=1}^h g^k$, where $h\le n$ and $g^1,g^2,\ldots ,g^h\in {\mathcal {F}}(W)$ are elementary vectors that conform to *z*. Further, for each $i=1,2,\ldots ,h-1$, $\textrm{supp}(g^i)\setminus \cup _{j=i+1}^h \textrm{supp}(g^j)\ne \emptyset$. A fundamental result on elementary vectors asserts the existence of a conformal circuit decomposition, see e.g. [9, 16]. Note that there may be multiple conformal circuit decompositions of the same vector.

### Lemma 3.2

For every subspace $W\subseteq {\mathbb {R}}^n$, every $z\in W$ admits a conformal circuit decomposition.

Given $A\in {\mathbb {R}}^{m\times n}$, we define the extended subspace ${\mathcal {X}}_A\subset {\mathbb {R}}^{n+m}$ as ${\mathcal {X}}_A{:}{=}\ker (A\mid -I_m)$. Hence, for every $z\in {\mathbb {R}}^n$, $(z,Az)\in {\mathcal {X}}_A$. For $z\in {\mathbb {R}}^n$, a *generalized path-circuit decomposition of* *z* *with respect to* *A* is a decomposition $z=\sum _{k=1}^h g^k$, where $h\le n+m$, and for each $k\in [h]$, $(g^k,Ag^k)\in {\mathbb {R}}^{n+m}$ is an elementary vector in ${\mathcal {X}}_A$ that conforms to (*z*, *Az*). Note that this corresponds to a conformal circuit decomposition of (*z*, *Az*) in ${\mathcal {X}}_A$. We say that $g^k$ is an *inner vector* in the decomposition if $Ag^k=0$ and an *outer vector* otherwise.

### Definition 3.3

We say that $z\in {\mathbb {R}}^n$ is *cycle-free with respect to* *A*, if no $y\in \ker (A)$, $y\ne 0$ exists that conforms *z*.

Note that being cycle-free is equivalent to the property that all generalized path-circuit decompositions of *z* contain outer vectors only. The following lemma will play a key role in analyzing our algorithms.

### Lemma 3.4

Let $A\in {\mathbb {R}}^{m\times n}$ and let $z\in {\mathbb {R}}^n$ be cycle-free with respect to *A*. Then

$$\begin{aligned} \Vert z\Vert _\infty \le \kappa ({{\mathcal {X}}_A})\cdot \Vert Az\Vert _1\, \quad \text{ and }\quad \Vert z\Vert _2\le m\cdot \kappa ({{\mathcal {X}}_A})\cdot \Vert Az\Vert _2\, . \end{aligned}$$

### Proof

Consider a generalized path-circuit decomposition $z=\sum _{k=1}^h g^k$. Since *z* is cycle-free, for each $g^k$, $Ag^k\ne 0$, and therefore $|g^k_j|\le \kappa ({{\mathcal {X}}_A})\cdot |(Ag^k)_i|$ for any $j\in \textrm{supp}(g^k)$ and $i\in \textrm{supp}(Ag^k)$. By the conformity property, $|z_j|=\sum _{k=1}^h |g^k_j|$ for $j\in [n]$ and $|(Az)_i|=\sum _{k=1}^h|(Ag^k)_i|$ for $i\in [m]$. Thus, for any $j\in [n]$,

$$\begin{aligned} |z_j|=\sum _{k=1}^h |g^k_j|\le \kappa ({{\mathcal {X}}_A})\cdot \sum _{i=1}^m \sum _{k=1}^h |(Ag^k)_i|=\kappa ({{\mathcal {X}}_A})\cdot \sum _{i=1}^m |(Az)_i|=\kappa ({{\mathcal {X}}_A})\cdot \Vert Az\Vert _1\, . \end{aligned}$$

For the second inequality, note that $\Vert g^k\Vert _2\le \sqrt{m}\cdot \kappa ({{\mathcal {X}}_A})|(Ag^k)_i|$ for any $k\in [h]$ and $i\in \textrm{supp}(Ag^k)$, since for any elementary vector $(g^k,Ag^k)\in {\mathcal {X}}_A$ with $\textrm{supp}(Ag^k)\ne 0$, the columns in $\textrm{supp}(g^k)$ must be linearly independent, and therefore $|\textrm{supp}(g^k)|\le m$. This implies

$$\begin{aligned} \Vert z\Vert _2\le \sum _{k=1}^h \Vert g^k\Vert _2\le \sqrt{m}\cdot \kappa ({{\mathcal {X}}_A})\cdot \Vert Az\Vert _1\le m\cdot \kappa ({{\mathcal {X}}_A})\cdot \Vert Az\Vert _2\, . \end{aligned}$$

$$\square $$

The following lemma is trivial for the input matrix since we assume $\Vert A\Vert _1 \ge 1$. However, we also need this guarantee for its column submatrices in the recursive calls.

### Lemma 3.5

For any non-zero matrix $A\in {\mathbb {R}}^{m\times n}$, $\kappa ({\mathcal {X}}_A) \Vert A\Vert _1 \ge 1$.

### Proof

Let $e_i \in {\textbf{R}}^n$ be the standard basis vector with a 1 in the *i*-th position and zeros elsewhere. Then, $(e_i, Ae_i)$ is an elementary vector in ${\mathcal {X}}_A$, which implies $\kappa ({\mathcal {X}}_A) \ge 1/|A_{ij}|$ for every *j* with $A_{ij} \ne 0$. Consequently, we have $\kappa ({\mathcal {X}}_A) \Vert A\Vert _1 \ge 1$. $\square$

When *A* is clear from the context, we simply use $\kappa =\kappa ({\mathcal {X}}_A)$. If *A* is a node-arc incidence matrix of a directed graph, then *A*, and consequently also $(A\mid -I_m)$ is a TU matrix, implying ${\bar{\kappa }}({\mathcal {X}}_A)=1$. For undirected graph incidence matrices, one can show ${\bar{\kappa }}({\mathcal {X}}_A)\le 2$.

### Lemma 3.6

Let $A\in {\mathbb {R}}^{n\times m}$. Then $\theta _{1,\infty }(A)\le \kappa ({\mathcal {X}}_A)$ and $\theta _{2,2}(A)\le m\cdot \kappa ({\mathcal {X}}_A)$.

### Proof

We need to show that for any $x\in [{\textbf{0}},u]$, and any $b\in {\mathbb {R}}^m$, whenever ${\mathcal {P}}_{A,b,u}$ is nonempty, there exists an $\bar{x}\in {\mathcal {P}}_{A,b,u}$ such that $\Vert {{\bar{x}}}-x\Vert _\infty \le \kappa ({\mathcal {X}}_A) \Vert Ax-b\Vert _1$ and $\Vert {{\bar{x}}}-x\Vert _2\le m\cdot \kappa ({\mathcal {X}}_A) \Vert Ax-b\Vert _2$. Let us select ${{\bar{x}}}\in {\mathcal {P}}_{A,b,u}$ as the nearest feasible point to *x* in $\ell _2$-norm; in particular, $A{{\bar{x}}}= b$. We claim that ${{\bar{x}}}-x$ is cycle-free with respect to *A*. Indeed, if a generalized path-circuit decomposition of ${{\bar{x}}}-x$ contained an inner vector $g^k$, then ${{\bar{x}}}'=x+g^k$ would also be feasible, with $\Vert {{\bar{x}}}'-x\Vert _2<\Vert {{\bar{x}}}-x\Vert _2$. Thus, Lemma 3.4 can be applied with $z=x - {{\bar{x}}}$ and the claims follow. $\square$

The first inequality may be tight. Assume there exists an elementary vector (*g*, *Ag*) in ${\mathcal {X}}_A$ such that $|g_i|=\kappa ({\mathcal {X}}_A)$ for all $i\in \textrm{supp}(g)$, $|\textrm{supp}(Ag)|=1$, and $(Ag)_i=1$ for the nonzero component. Further, let $b=0$, and let $u_i=0$ for all $i\notin \textrm{supp}(g)$. Since (*g*, *Ag*) is a support minimal nonzero vector in ${\mathcal {X}}_A$, it follows that the only feasible solution to $A{{\bar{x}}}=0$, ${{\bar{x}}}\in [{\textbf{0}},u]$ is ${{\bar{x}}}=0$. Thus, we get a tight example with $\theta _{1,\infty }(A)= \kappa ({\mathcal {X}}_A)$. The same example shows tightness of the second inequality up to a factor $\sqrt{m}$.

Our algorithm will also find dual certificates. The next lemma shows that the corresponding dual systems have the same circuit imbalances.

### Lemma 3.7

For any matrix $A\in {\mathbb {R}}^{m\times n}$, $\kappa (A^\top | I_n)=\kappa ({\mathcal {X}}_A)$.

### Proof

Recall that ${\mathcal {X}}_A=\ker (A|-I_m)$. It is easy to verify that $\ker (I_n | A^\top )$ is the orthogonal complement of ${\mathcal {X}}_A$. The statement then follows by Lemma 3.1, noting also that reordering the columns does not change the circuit imbalances. $\square$

### Guessing the circuit imbalances

Given a matrix $A\in {\mathbb {R}}^{m\times n}$, there is no hope of getting any reasonable approximation of the circuit imbalance values. It is NP-hard to approximate $\kappa (A)$ within a factor $2^{\textrm{poly}(m)}$ for $A\in {\mathbb {R}}^{m\times n}$, see [5], using a result of Tunçel [20] on the related condition number ${\bar{\chi }}(A)$. However, our algorithms make use of the values $\kappa (A)$ and ${\bar{\kappa }}(A)$.

Nevertheless, one can use a guessing procedure that guarantees the same asymptotic running times without knowing these values. First, note that upper bounds rather than exact values suffice throughout. Algorithm 1 below makes recursive calls to column submatrices of the original input matrix *A*. If ${\bar{\kappa }}(A)\le {\hat{\kappa }}$ for the input matrix *A*, then ${\hat{\kappa }}$ is an upper bound on all the circuit imbalance values seen in the recursive calls.

As our initial estimate, we set ${\hat{\kappa }}=1$. When run with a correct guess ${\hat{\kappa }} \ge {\bar{\kappa }}(A)$, Algorithm 1 (see Sect. 5) returns approximately optimal primal and dual solutions to LP(*A*, *b*, *c*, *u*). Running it with an incorrect guess may lead to a failure while running the algorithm: either R-FGM does not find a solution of the required accuracy within the given number of steps, or the final primal and dual solutions do not satisfy the required approximate feasibility and complementarity properties. We can easily detect both kind of failures. If no failure is detected, then the primal and dual solutions certify approximate optimality for each other; this may happen even when ${\hat{\kappa }}<{\bar{\kappa }}(A)$. Each time we detect a failure, we double the estimate ${\hat{\kappa }}$ and restart the algorithm.

The overall running time bound in Theorem 1.1 is also the total running time of this process, because at each call we have ${\hat{\kappa \le }} 2{\bar{\kappa }}(A)$, and the running time bound of the final run dominates the running time bound of all previous runs.

## Main ideas and key statements

Before describing the algorithm in Sect. 5, we highlight the key ideas and formulate the main underlying proximity results. We gradually reduce LP(*A*, *b*, *c*, *u*) by fixing some variables to their upper or lower bounds, and replacing the cost vector by an equivalent one of smaller norm. We first start by describing the simpler feasibility algorithm. The optimization algorithm has two components: the outer loop and the inner loop.

### The feasibility algorithm

We first show how the R-FGM algorithm from [15] leads to a simple algorithm for finding a $\delta$-feasible solution. Here, we assume that the LP is feasible. In the proof of Theorem 1.1 in Sect. 5.3, we explain how this assumption can be removed in general.

#### Theorem 4.1

There is an algorithm Feasible $(A,b,u,\delta )$, which, on input $A\in {\mathbb {R}}^{m\times n}$, $\Vert A\Vert _1\ge 1$, $b\in {\mathbb {R}}^m$, $u\in {\mathbb {R}}^n$, supposing the system $Ax=b$, $x\in [{\textbf{0}},u]$ is feasible, finds a $\delta$-feasible solution using $O\big (m \Vert A\Vert _2 \cdot \kappa ({\mathcal {X}}_A)\cdot \log (m \Vert b\Vert _1 / \delta )\big )$ iterations of R-FGM.

#### Proof

Let $f(x)=\frac{1}{2}\Vert Ax-b\Vert ^2$. We use the R-FGM algorithm from [15] to find an $\varepsilon$-approximate minimizer of *f*(*x*) over $x\in [{\textbf{0}},u]$, i.e., the system (1), where $\varepsilon {:}{=}\Vert A\Vert _1^2 \cdot \delta ^2 / (2m)$. We choose $x=0$ as the starting point. By Theorem 2.5, $O(\Vert A\Vert _2 \cdot \theta _{2,2}(A)\cdot \log (m \Vert b\Vert _1/ \delta ))$ iterations suffice (using $\Vert A\Vert _1\ge 1$). The bound on the number of iterations follows, for by Lemma 3.6, $\theta _{2,2}(A) \le m \cdot \kappa ({\mathcal {X}}_A)$.

By the assumption that the system is feasible, the optimum value is 0. Thus, an $\varepsilon$-approximate solution has $f(x)\le \varepsilon$, which yields a $\delta$-feasible solution. $\square$

### The outer loop

In the outer loop, our goal is to find a $\delta ^\textrm{feas}$-feasible and $\delta ^\textrm{opt}$-optimal solution to LP(*A*, *b*, *c*, *u*). We distinguish these two accuracy parameters for the sake of the recursive algorithm, where the required feasibility and optimality accuracies need to be changed differently in the recursive calls.

***Primal-dual optimality and cost shifting.***

We use primal-dual arguments, making variable fixing decisions based on approximate complementarity conditions. The dual to LP(*A*, *b*, *c*, *u*) can be written as 
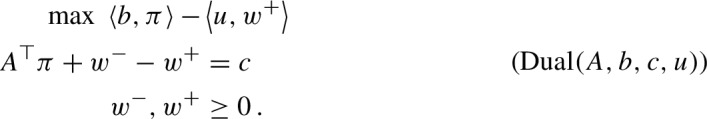
 Note that given $\pi \in {\mathbb {R}}^m$, the unique best choice of the variables $w^-$ and $w^+$ is $w^-=\max \{c-A^\top \pi ,0\}$ and $w^+=\max \{A^\top \pi -c,0\}$. When we speak of a dual solution $\pi \in {\mathbb {R}}^m$, we mean its extension with these variables. Recall the primal-dual optimality conditions: $x^*\in {\mathcal {P}}_{A,b,u}$ and $\pi \in {\mathbb {R}}^m$ are optimal respectively to LP(*A*, *b*, *c*, *u*) and to Dual(*A*, *b*, *c*, *u*) if and only if the following holds:

5 $$\begin{aligned} \text{ if } \, A_i^\top \pi <c_i \,\text{ then } \, x_i=0, \,\, \text{ and } \text{ if }\,\, A_i^\top \pi >c_i \,\,\text{ then }\,\, x_i=u_i \,\,\text{ for } \text{ every } \,\, i\in [n]. \end{aligned}$$

Also note that we can naturally shift the cost function for any $\pi \in {\mathbb {R}}^m$ as stated in the next lemma.

#### Lemma 4.2

 LP(*A*, *b*, *c*, *u*) has exactly the same solutions and the same optimum value as the following linear program:

6 $$\begin{aligned} \begin{aligned} \min&\left\langle c - A^\top \pi ,x\right\rangle + \left\langle b,\pi \right\rangle \, \quad \text{ s.t. }\quad Ax = b\, ,\quad x\in [{\textbf{0}},u]\, . \end{aligned} \end{aligned}$$

***Approximate complementarity and proximity.***

Assume now that we have a pair of primal and dual solutions *x* and $\pi$ that do not satisfy complementarity, but we have a quantitative bound on the violation. Namely, for a suitably chosen threshold $\sigma \ge 0$, let

$$\begin{aligned} \begin{aligned} \theta (x,\pi ,\sigma )&{:}{=}\sum _{c_{i} -A_i^\top \pi> \sigma }x_{i} +\sum _{c_{i} -A_i^\top \pi < -\sigma } (u_{i}-x_{i})\, ,\quad \text{ and } \\ J(\pi ,\sigma )&{:}{=}\left\{ i\in [n]:\, |c_{i} - A_i^\top \pi |>n\cdot \lceil \kappa ({\mathcal {X}}_A)\rceil \cdot \sigma \right\} \, . \end{aligned} \end{aligned}$$

Note that if *x* and $\pi$ are primal and dual optimal, then the primal-dual complementarity constraints (5) imply $\theta (x,\pi ,0)=0$. Let us assume that for some $\sigma >0$, this quantity is still small. Note also that $J(\pi ,\sigma )$ is the set of indices where the absolute value of the slack is much higher than the threshold $\sigma$. In particular, $\min \{x_i,u_i-x_i\}\le \theta (x,\pi ,\sigma )$ on these indices. Our key proximity result asserts that there exists an optimal solution that is close to the current solution on these indices. The proof is deferred to Sect. 6.

#### Lemma 4.3

Let $x\in {\mathcal {P}}_{A,b,u}$ be a feasible solution. Then there exists an optimal solution $x^*$ for LP(*A*, *b*, *c*, *u*) such that

$$\begin{aligned} |x_i-x^*_i|\le \kappa ({\mathcal {X}}_A) \cdot \theta (x,\pi ,\sigma ) \end{aligned}$$

for all $$i\in J(\pi ,\sigma )$$.

***Variable fixing.***

Assume that from the inner loop of the algorithm we get $x\in [{\textbf{0}},u]$ and $\pi \in {\mathbb {R}}^m$ such that the feasibility violation $\Vert Ax-b\Vert _1$ and the complementarity violation $\theta (x,\pi ,\sigma )$ are both tiny for the choice $\sigma {:}{=} \Vert c\Vert _{\infty } / (4 n \lceil \kappa ({\mathcal {X}}_A)\rceil )$. Note that the for this choice, the threshold in the definition of $J(\pi ,\sigma )$ becomes $\Vert c\Vert _\infty /4$. We partition $J(\pi ,\sigma )$ into

$$\begin{aligned} \begin{aligned} J_1&{:}{=}\left\{ i\in J(\pi ,\sigma ) ~\big |~ c_i-A_i^\top \pi <-\tfrac{\Vert c\Vert _\infty }{4}\right\} \, ,\quad \text{ and }\\J_2&{:}{=}\left\{ i\in J(\pi ,\sigma )~\big |~ c_i-A_i^\top \pi >\tfrac{\Vert c\Vert _\infty }{4}\,\right\} \, , \end{aligned} \end{aligned}$$

We apply Lemma 4.3 to the problem with the modified right hand side $b'=Ax$. By ensuring that $\theta (x,\pi ,\sigma )$ is sufficiently small, we will see that there is an optimal solution $x^*$ with $x^*_i\approx 0$ for $i\in J_1$ and $x_i^*\approx u_i$ for $i\in J_2$.

We fix these variables to the lower and upper bounds, respectively, and shift the cost function according to $\pi$. Thus, we specify the following new LP. Let $N{:}{=}[n]\setminus (J_1\cup J_2)$ and ${{\bar{b}}}{:}{=}A_{\scriptscriptstyle {\!N}}x_{\scriptscriptstyle {\!N}}$. 
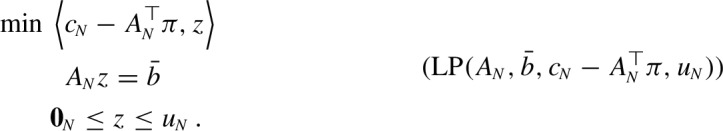
 We show the following result, which says the optimal solution of LP$(A_{\scriptscriptstyle {\!N}},{{\bar{b}}},c_{\scriptscriptstyle {\!N}}- A_{\scriptscriptstyle {\!N}}^\top \pi ,u_{\scriptscriptstyle {\!N}})$ provides an approximately feasible and optimal solution to LP(*A*, *b*, *c*, *u*). The approximation is in terms of $\theta (x,\pi ,\sigma )$ and $\Vert Ax-b\Vert _1$. Recall that $\Phi (A,b,c,u)$ denotes the optimal value, the value achieved by the solution to LP(*A*, *b*, *c*, *u*). The proof is given in Sect. 6.

#### Theorem 4.4

For $A\in {\mathbb {R}}^{m\times n}$, $b\in {\mathbb {R}}^m$, and $c,u\in {\mathbb {R}}^n$ such that LP(*A*, *b*, *c*, *u*) is feasible, let $\sigma {:}{=}\Vert c\Vert _\infty /(4n\cdot \lceil \kappa ({\mathcal {X}}_A)\rceil )$, and let $x\in [{\textbf{0}},u]$ and $\pi \in {\mathbb {R}}^m$ be a pair of (not necessarily feasible) primal and dual solutions. Then, LP$(A_{\scriptscriptstyle {\!N}},{{\bar{b}}},c_{\scriptscriptstyle {\!N}}- A_{\scriptscriptstyle {\!N}}^\top \pi ,u_{\scriptscriptstyle {\!N}})$ is feasible and, in addition satisfies the following:

- feasibility condition:
  7 $$\begin{aligned} \Vert b-{{\bar{b}}}-A_{\scriptscriptstyle {\!J_2}}u_{\scriptscriptstyle {\!J_2}}\Vert _1\le \theta (x,\pi ,\sigma )\cdot \Vert A\Vert _1+\Vert Ax-b\Vert _1\, , \end{aligned}$$
- optimality condition:
  8 $$\begin{aligned} \nonumber&\big |\Phi (A_{\scriptscriptstyle {\!N}},{{\bar{b}}},c_{\scriptscriptstyle {\!N}}- A_{\scriptscriptstyle {\!N}}^\top \pi ,u_{\scriptscriptstyle {\!N}}) + \left\langle {{\bar{b}}},\pi \right\rangle +\left\langle c_{\scriptscriptstyle {\!J_2}},u_{\scriptscriptstyle {\!J_2}}\right\rangle -\Phi (A,b,c,u)\big | \nonumber \\&\quad = \big |\Phi (A_{\scriptscriptstyle {\!N}},{{\bar{b}}},c_{\scriptscriptstyle {\!N}},u_{\scriptscriptstyle {\!N}})+\left\langle c_{\scriptscriptstyle {\!J_2}},u_{\scriptscriptstyle {\!J_2}}\right\rangle -\Phi (A,b,c,u)\big |\nonumber \\&\quad \le \kappa ({\mathcal {X}}_A)\cdot \Vert c\Vert _1\cdot \Vert Ax-b\Vert _1\nonumber \\&\qquad \phantom {\le {}} + |J_1\cup J_2|\cdot \kappa ({\mathcal {X}}_A)\cdot \Vert c\Vert _{1}\cdot \big (2 + \kappa ({\mathcal {X}}_A) \Vert A\Vert _1\big )\cdot \theta (x,\pi ,\sigma )\, , \end{aligned}$$
- cost reduction: $$\Vert c_N-A_N^\top \pi \Vert _\infty \le \Vert c\Vert _\infty /4$$.

With this theorem, if one can find a pair $(x, \pi )$ such that the right hand sides of inequalities (7) and (8) are tiny, then LP(*A*, *b*, *c*, *u*) can be reduced to LP$(A_{\scriptscriptstyle {\!N}},{{\bar{b}}},c_{\scriptscriptstyle {\!N}}- A_{\scriptscriptstyle {\!N}}^\top \pi ,u_{\scriptscriptstyle {\!N}})$ with a tiny loss on feasibility and optimality. Moreover, each iteration reduces the $\ell _\infty$-cost on the remaining variables by a factor 4. One can repeat this procedure and ultimately reduce the original problem to one with an extremely small objective function value and possibly with fewer variables. Solving this problem will give a good enough solution to the original LP(*A*, *b*, *c*, *u*), after restoring any variables fixed to the lower or upper bounds.

It is possible that both $J_1$ and $J_2$ are empty. This means that $\Vert c-A^\top \pi \Vert _\infty \le \Vert c\Vert _\infty / 4$; we can simply recurse with the same *b* but improved cost function. Note that we could make progress more agressively by a preprocessing step that projects *c* to the kernel of *A*; this gets a cost vector of the form $c'=c-A^\top \pi$ with the smallest possible $\ell _2$-norm—such a preprocessing is used in the strongly polynomial algorithms [4, 18, 19]. Setting a slightly smaller $\sigma$ would then guarantee variable fixing in every iteration. However, the projection amounts to solving a system of linear equations that is computationally more expensive. We instead proceed with lazier updates as above.

### The inner loop

Next, we describe our approach for obtaining a pair $(x, \pi )$ such that the right hand sides of inequalities (7) and (8) are tiny, which is the purpose of the inner loop. For this, we need to guarantee that $\theta (x,\pi ,\sigma )$ and $\Vert Ax-b\Vert _1$ are sufficiently small. We use a potential function $F_{\tau }(x)$ of the form (3) for a modified cost function ${\hat{c}}$.

As noted in the introduction, if ${\hat{c}}=c/\Vert c\Vert _\infty$, and $\tau$ is within $\delta /2$ of the optimum value of LP(*A*, *b*, *c*, *u*), then a $\delta '$-approximate minimizer to $F_{\tau }(x)$ for a suitably chosen $\delta '$ would immediately give a $\delta$-approximate and $\delta$-feasible solution to LP(*A*, *b*, *c*, *u*). Thus, we would not need the outer loops; a binary search on $\tau$ and using the feasibility algorithm on this system would already give the desired solution, without the need for variable fixings in the outer loop.

However, the Hoffman-constant corresponding to the function (3) with ${\hat{c}}=c/\Vert c\Vert _\infty$ could be unbounded in terms of ${\bar{\kappa }}({\mathcal {X}}_A)$ if *c* can be arbitrary, as discussed in Sect. 10. To circumvent this problem, we discretize $c/\Vert c\Vert _\infty$ into integer multiples of $\varepsilon =1/(8n\cdot \lceil \kappa ({\mathcal {X}}_A)\rceil )=\sigma /(2\Vert c\Vert _\infty )$.

Using the discretized ${\hat{c}}$, for a suitable choice of $\tau$, we can guarantee (7) and (8), that is, bound $\Vert Ax-b\Vert _1$ and $\theta (x,\pi ,\sigma )$, where the dual solution $\pi$ is defined based on the gradient of $F_{\tau }(x)$ as $\pi {:}{=}\frac{\Vert c\Vert _{\infty }}{\Vert A\Vert ^2_1\alpha }(b-Ax)$ for $\alpha {:}{=}\max \{0, \langle {\hat{c}}, x \rangle - \tau \}$.

To bound the infeasibility $\Vert Ax-b\Vert _1$, we need to find a solution *x* where $F_{\tau }(x)$ is small, since $\Vert Ax-b\Vert _1\le (2m\Vert A\Vert _1^2 F_{\tau }(x))^{1/2}$. Therefore, $\tau$ should not be much smaller than the optimum value $\Phi (A,b,{\hat{c}},u)$. The bound on $\theta (x,\pi ,\sigma )$ can be shown by arguing that the improving directions of the gradient are small at an approximately optimal solution *x*: $x_i \approx 0$ if $\nabla _i F_{\tau }(x) \gg 0$ and $x_i \approx u_i$ if $\nabla _i F_{\tau }(x) \ll 0$, and that $|c_i /\Vert c\Vert _\infty -{\hat{c}}_i| \cdot \Vert c\Vert _\infty \le \Vert c\Vert _\infty \cdot \varepsilon =\sigma /2$. We also need that $\alpha >0$ and is not too small. Based on these requirements, we can establish a narrow (but not too narrow) interval of $\tau$ where a sufficiently accurate approximate solution to $F_{\tau }(x)$ exists. Using that $F_{\tau }^\star {:}{=} \min \{F_{\tau }(x)\mid x\in [{\textbf{0}},u]\}$ is a Lipschitz-continuous and non-increasing continuous function in $\tau$, we can find a suitable $\tau$ by binary search.

### Dual certificates

As discussed above, our goal is not just to find a $\delta ^\textrm{feas}$-feasible and $\delta ^\textrm{opt}$-optimal solution, but also a dual certificate for the latter property. This is important as it enables us to verify the correctness of the solution, which is only guaranteed when using an estimate ${\hat{\kappa \ge {\bar{\kappa }}}}$.

#### Definition 4.5

Let $A\in {\mathbb {R}}^{m\times n}$, $b\in {\mathbb {R}}^m$, $c,u\in {\mathbb {R}}^n$ and $\delta \ge 0$, and let $x\in [{\textbf{0}},u]$. We say that $(\pi ,w^+,w^-)\in {\mathbb {R}}^{m\times n\times n}$ is a $\delta$-*certificate for* *x*, if

(i) $$A^\top \pi +w^--w^+=c$$,
(ii) $0\le w_i^-\le 2\delta \Vert c\Vert _\infty /x_i$, $0\le w_i^+\le 2\delta \Vert c\Vert _\infty /(u_i-x_i)$ for all $i\in [n]$, and
(iii) $$\Vert \pi \Vert _\infty \le 2\delta \Vert c\Vert _\infty /\Vert Ax-b\Vert _1$$.

Our next lemma shows that $\delta$-certificates indeed certify approximate optimality, and conversely, for every approximately optimal solution, such a certificate can be found. The proof is given in Sect. 9.

#### Lemma 4.6

Let $A\in {\mathbb {R}}^{m\times n}$, $b\in {\mathbb {R}}^m$, $c,u\in {\mathbb {R}}^n$, and let $x\in [{\textbf{0}},u]$.

(i) If there is a $\delta$-certificate for *x* for some $\delta \ge 0$, then *x* is $(4n+2)\delta$-optimal.
(ii) Suppose $0\le \delta ^\textrm{feas}\cdot n\cdot \kappa ({\mathcal {X}}_A) \cdot \Vert A\Vert _1 \le \delta ^\textrm{opt}$. If *x* is a $\delta ^\textrm{feas}$-feasible and $\delta ^\textrm{opt}$-optimal solution, then there exists a $\delta ^\textrm{opt}$-certificate for *x*.

Our next theorem justifies this concept and provides certificates efficiently. The proof is also deferred to Sect. 9.

#### Theorem 4.7

Suppose $A\in {\mathbb {R}}^{m\times n}$, $b\in {\mathbb {R}}^m$, $x,c,u\in {\mathbb {R}}^n$, $0 \le \delta ^\textrm{feas}\cdot n\cdot \kappa ({\mathcal {X}}_A) \cdot \Vert A\Vert _1 \le \delta ^\textrm{opt}$, $\Vert A\Vert _1 \ge 1$ and $x\in [{\textbf{0}},u]$ is both $\delta ^\textrm{feas}$-feasible and $\delta ^\textrm{opt}$-optimal. Then there is an algorithm Dual-Certificate $(x,A,b,c,u,\delta ^\textrm{feas},\delta ^\textrm{opt})$ which on such inputs finds a $2\cdot \delta ^\textrm{opt}$-certificate for *x* in $O\big (m \sqrt{n} \cdot \Vert A\Vert _1 \cdot \kappa ({\mathcal {X}}_A)\cdot \log (n \Vert c\Vert _1 / \delta ^\textrm{opt})\big )$ iterations of R-FGM.

## The algorithm

We describe our algorithm under the simplifying assumption that the exact values of $\kappa (A)$ and ${\bar{\kappa }}(A)$ are known for the input matrix as well as all submatrices obtained by column deletions. As discussed in Sect. 3.1, even though these quantities cannot be computed, one can obtain the same asymptotic running time bounds by repeatedly guessing an estimate ${\hat{\kappa }}$.

### The outer loop: variable fixing

Algorithm 1 takes as input (*A*, *b*, *c*, *u*) such that LP(*A*, *b*, *c*, *u*) is feasible, and accuracy parameters $\delta ^\textrm{feas}$ and $\delta ^\textrm{opt}$ such that $0\le \delta ^\textrm{feas}\cdot 8 n \sqrt{m} \cdot \kappa ({\mathcal {X}}_A)\cdot \Vert A\Vert _1 \le \delta ^\textrm{opt}$.

No feasibility assumption is made in Theorem 1.1; we prove this theorem in Sect. 5.3. We handle feasibility in the same spirit as in Simplex, using a two-stage approach. Algorithm 1 uses a subroutine ${\texttt {GetPrimalDualPair}}(A,b,c,u,\delta ^\textrm{feas},\delta ^\textrm{opt})$ specified as follows.
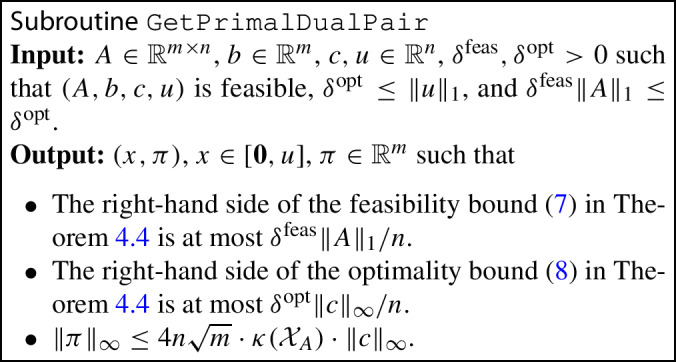


**Algorithm 1:**
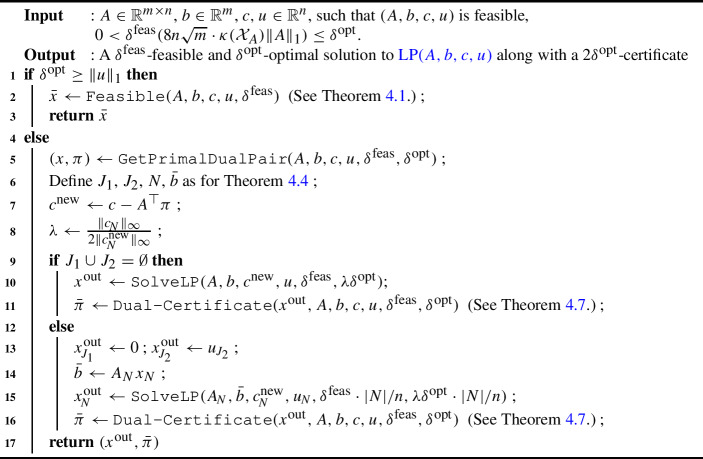
$$\texttt {SolveLP}$$

The following theorem provides the analysis of the outer loop. The proof is deferred to Sect. 7.

#### Theorem 5.1

If LP(*A*, *b*, *c*, *u*) is feasible, then Algorithm 1 returns a $\delta ^\textrm{feas}$-feasible solution that is $\delta ^\textrm{opt}$-optimal along with a $2 \delta ^\textrm{opt}$-certificate. It makes at most $\log _2(n\Vert u\Vert _1/\delta ^\textrm{opt})$ many recursive calls.

### The inner loop: fast gradient with binary search

We now describe $\texttt {GetPrimalDualPair}(A,b,c,u,\delta ^\textrm{feas},\delta ^\textrm{opt})$, introduced at the beginning of Sect. 5.1. The subroutine needs to output primal and dual vectors $(x,\pi )$ satisfying the feasibility and optimality bounds in Theorem 4.4. In particular, we need to bound $\theta (x, \pi , \sigma ) = \sum _{i : c_i - A_i^\top \pi > \sigma } x_i + \sum _{i: c_i - A_i^\top \pi < - \sigma } (u_i - x_i)$ and $\Vert A x - b\Vert _1$ for $\sigma =\Vert c\Vert _\infty /(4n\cdot \lceil \kappa ({\mathcal {X}}_A)\rceil )$. Let us define the accuracy parameter

9 $$\begin{aligned} \varepsilon {:}{=} \frac{1}{8n\cdot \lceil \kappa ({\mathcal {X}}_A)\rceil }=\frac{\sigma }{2\Vert c\Vert _\infty }\, . \end{aligned}$$

Thus, $1/\varepsilon$ is integer. For some parameter $\tau \in {\mathbb {R}}$, we use the potential function

10 $$\begin{aligned} F_{\tau }(x) {:}{=} \frac{1}{2}(\max \{0, \langle {\hat{c}}, x \rangle - \tau \})^2 + \frac{1}{2\Vert A\Vert _1^2} \Vert Ax - b\Vert _2^2\, , \end{aligned}$$

where the rounded cost function ${\hat{c}}$ is defined by taking the normalized vector $c/\Vert c\Vert _\infty$, and rounding each entry to the nearest integer multiple of $\varepsilon$ in the direction of the 0 value (i.e., rounding down the positive entries and rounding up the negative entries). Recalling that $1/\varepsilon$ is an integer, we have $\Vert {\hat{c}}\Vert _\infty = 1$.

Let $F_{\tau }^\star {:}{=} \min \{F_{\tau }(x)\mid x\in [{\textbf{0}},u]\}$ denote the optimum value. We say that $x\in [{\textbf{0}},u]$ is a $\zeta$-*approximate minimizer* of $F_{\tau }$ if $F(x)\le F_{\tau }^\star + \zeta$. The following proposition is immediate.

#### Proposition 5.2

$F_{\tau }^\star$ is a non-increasing continuous function of $\tau$. If LP$(A,b,{\hat{c}},u)$ is feasible, then $\Phi (A, b, {\hat{c}}, u)$ is the smallest value of $\tau$ such that $F_{\tau }^\star = 0$.

The main driver of our algorithm is Necoara, Nesterov, and Glineur’s R-FGM algorithm, applied to $F_{\tau }$. We specify this subroutine as follows.
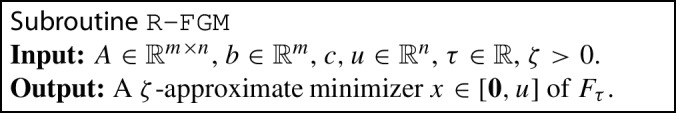


The purpose of $\texttt {GetPrimalDualPair}{}$ is to identify a value $\tau$ by binary search that is slightly below $\Phi (A,b,{\hat{c}},u)$. We show that there is a suitable $\tau$ such that a sufficiently accurate approximate minimizer of $F_{\tau }$ returns the required primal solution *x*. Moreover, we can also construct the dual $\pi$ from the gradient of $F_{\tau }$ at this point.

We define some further parameters to calibrate the accuracy used in the algorithm.

11 $$\begin{aligned} {\mathcal {C}}&{:}{=} n\sqrt{m}\cdot \kappa ({{\mathcal {X}}_A}) \cdot \Vert A\Vert _1\, ,\nonumber \\ \overline{{\mathcal {C}}}&{:}{=} 64n {\mathcal {C}}\cdot \kappa ({\mathcal {X}}_A)=64 n^2 \sqrt{m} \cdot \kappa ^2({\mathcal {X}}_A) \cdot \Vert A\Vert _1\, ,\nonumber \\ \zeta&{:}{=} {\Big (\frac{\delta ^\textrm{feas}}{4 \kappa ^2({\mathcal {X}}_A)n^4 \overline{{\mathcal {C}}}\sqrt{m} }\Big )^2}\, . \end{aligned}$$

These admit the following simple lower bounds.

#### Lemma 5.3

$${\mathcal {C}}\ge n\sqrt{m}$$, $$\overline{{\mathcal {C}}}\ge 64n\sqrt{m}$$, and $$\overline{{\mathcal {C}}}\sqrt{\zeta } = {\frac{\delta ^\textrm{feas}}{4n^4\sqrt{m}\cdot \kappa ^2({\mathcal {X}}_A)}}$$.

#### Proof

The first bound follows by Lemma 3.5, and the second bound from the first and using $\kappa ({\mathcal {X}}_A)\ge 1$. The third bound is immediate from the definition. $\square$

Algorithm $\texttt {GetPrimalDualPair}$ is shown in Algorithm 2.

**Algorithm 2:**
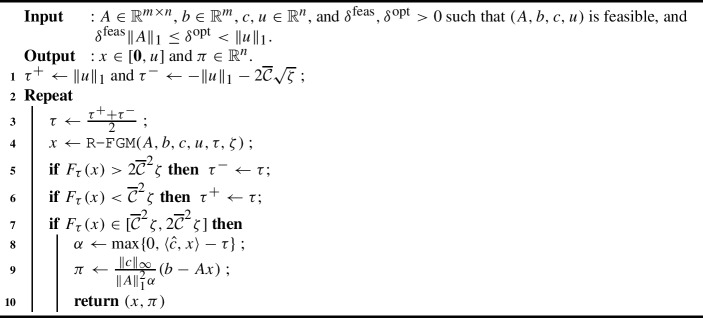
$$\texttt {GetPrimalDualPair}$$

#### Theorem 5.4

Assume LP(*A*, *b*, *c*, *u*) is feasible. Algorithm 2 makes $O\big (\log \big [\Vert u\Vert _1 nm\cdot \kappa ({\mathcal {X}}_{A})/\delta ^\textrm{feas}\big ]\big )$ calls to R-FGM, and altogether these calls use $O\big ( n^{1.5} m^2 \Vert A\Vert ^2_1 \cdot {\bar{\kappa }}^3({\mathcal {X}}_{A}) \log ^2 \big [\Vert u\Vert _1 n m \cdot \Vert A\Vert _1 \kappa ({\mathcal {X}}_A)/\delta ^\textrm{feas}\big ]\big )$ iterations. On terminating, it outputs $(x, \pi )$ satisfying:

(i) $$\theta (x, \pi , \sigma ) \cdot \Vert A\Vert _1 + \Vert Ax - b\Vert _1 \le \delta ^\textrm{feas}\Vert A\Vert _1 / n$$.
(ii) $$\kappa ({\mathcal {X}}_A)\cdot \Vert c\Vert _1\cdot \Vert Ax-b\Vert _1 + |J_1\cup J_2|\cdot \kappa ({\mathcal {X}}_A)\cdot \Vert c\Vert _{1}\cdot \big (2 + \kappa ({\mathcal {X}}_A) \Vert A\Vert _1\big )\cdot \theta (x,\pi ,\sigma ) \le \delta ^\textrm{opt}\Vert c\Vert _\infty / n $$.
(iii) $$\Vert \pi \Vert _{\infty } \le 4n \sqrt{m} \cdot \kappa ({\mathcal {X}}_A)\cdot \Vert c\Vert _{\infty } $$.

### Putting everything together

We now combine the above ingredients to prove Theorem 1.1.

#### Proof of Theorem 1.1

First, let us assume that LP(*A*, *b*, *c*, *u*) is feasible. We set $\delta ^\textrm{opt}= \delta / (8 n + 4)$ and start with the guess $\hat{\kappa }= 1$. Then, we set $\delta ^\textrm{feas}= \delta ^\textrm{opt}/ (8 n \sqrt{m} {\hat{\kappa }} \Vert A\Vert _1)$, and run the algorithm $\texttt {SolveLP}(A, b, c, u, \delta ^\textrm{feas}, \delta ^\textrm{opt})$. If it succeeds and outputs a primal solution *x* and a certificate $\pi$, then we can check if the solution is a $\delta ^\textrm{feas}$-feasible solution by checking the constraint $\Vert Ax - b\Vert _1 \le \delta ^\textrm{feas}\cdot \Vert A\Vert _1$; and check if $\pi$ is a $2 \delta ^\textrm{opt}$-certificate by checking the constraints in Definition 4.5. If *x* is a $\delta ^\textrm{feas}$-feasible solution and $\pi$ is a $2 \delta ^\textrm{opt}$-certificate, then we output the *x*. Otherwise, we double the value of ${\hat{\kappa }}$ and restart this procedure.

Assuming ${\hat{\kappa }} \ge {\bar{\kappa }}({\mathcal {X}}_A)$, $\texttt {SolveLP}(A, b, c, u, \delta ^\textrm{feas}, \delta ^\textrm{opt})$ returns a $\delta ^\textrm{feas}$-feasible and $\delta ^\textrm{opt}$-optimal solution along with a $2 \delta ^\textrm{opt}$-certificate. These provide a $\delta$-feasible and $\delta$-optimal solution, along with a dual certificate of $\delta$-optimality, in accordance with Lemma 4.6(i).

For the running time, $\texttt {SolveLP}(A, b, c, u, \delta ^\textrm{feas}, \delta ^\textrm{opt})$ makes at most $\log _2(n\Vert u\Vert _1/\delta ^\textrm{opt})$ recursive calls (Theorem 5.1), and, for each recursive call, $\texttt {GetPrimalDualPair}$ uses at most $O\left( n^{1.5} m^2 \Vert A\Vert _1^2 {{\hat{\kappa }}}^3 \right.$ $\log ^2 \left. \left( \Vert u\Vert _1 n m {{\hat{\kappa }}}/\delta ^\textrm{feas}\right) \right)$ iterations (Theorem 5.4). Note that ${\hat{\kappa }}$ will stop doubling no later than the first time ${\hat{\kappa }} \ge \bar{\kappa }({\mathcal {X}}_A)$. Therefore, the total number of iterations is at most $O\big ( n^{1.5} m^2 \Vert A\Vert _1^2 \cdot {{\bar{\kappa }}({\mathcal {X}}_A)}^3 \log ^3 \left( \Vert u\Vert _1 n m \Vert A\Vert _1 \cdot {\bar{\kappa }({\mathcal {X}}_A)}/\delta \big )\right)$ as $\delta = \delta ^\textrm{feas}/ [8 n \sqrt{m} {\hat{\kappa }} \Vert A\Vert _1 (8 n + 4)]$.

We now remove the feasibility assumption. We use a two-stage approach, similarly to Simplex. We consider the following extended system; ${\textbf{1}}\in {\mathbb {R}}^m$ denotes the all 1’s vector.

$$\begin{aligned} \begin{aligned} \min \left\langle {\textbf{1}},s'\right\rangle +&\left\langle {\textbf{1}},s''\right\rangle \\ Ax+s'-s''&=b\, \\ {\textbf{0}}\le x&\le u\\ {\textbf{0}}\le s',s''&\le \Vert b\Vert _\infty \end{aligned} \end{aligned}$$

This system is trivially feasible with the solution $s'_i=\max \{b_i,0\}$ and $s''_i=\max \{-b_i,0\}$. Moreover, denoting the constraint matrix as $B=(A\, |\, I_m\, |\, -I_m)$, note that $\kappa ({\mathcal {X}}_B)=\kappa ({\mathcal {X}}_A)$ and ${\bar{\kappa }}({\mathcal {X}}_B)={\bar{\kappa }}({\mathcal {X}}_A)$.

We obtain a $\delta / 4$-feasible and $\delta / 4$-optimal solution $({\bar{x}},s',s'' )$ for this system by applying $\texttt {SolveLP}$. If the original system was feasible, then $({\bar{x}},s',s'' )$ provides a solution to the new LP with objective value of at most $\delta / 4$. As the new cost vector is the all ones vector, we see that $\Vert s'\Vert _1+\Vert s''\Vert _1 < \delta /4$. As we show next, this implies that the returned solution ${{\bar{x}}}$ will be $\delta / 2$-feasible for the original system:

$$\Vert A {{\bar{x}}} - b\Vert _1 \le \Vert A {{\bar{x}}} + s' - s''-b\Vert _1 + \Vert s'\Vert _1 + \Vert s''\Vert _1 \le (\delta / 4)\cdot \Vert A\Vert _1 + \delta / 4 \le (\delta / 2)\cdot \Vert A\Vert _1.$$

Both the second and third inequalities use $\Vert A\Vert _1 \ge 1$. We now run the above algorithm with the modified right hand side $\bar{b}=A{{\bar{x}}}$. Now, $\textrm{LP}(A,{{\bar{b}}},c,u)$ is feasible, and a $\delta ^\textrm{feas}$-feasible solution for this system will also be $\delta$-feasible for the original system, since $\delta ^\textrm{feas}+ \delta / 2 <\delta$. $\square$

## Proofs of the proximity statements

In this section, we prove Lemma 4.3 and Theorem 4.4.

### Lemma 4.3

Let $x\in {\mathcal {P}}_{A,b,u}$ be a feasible solution. Then there exists an optimal solution $x^*$ for LP(*A*, *b*, *c*, *u*) such that

$$\begin{aligned} |x_i-x^*_i|\le \kappa ({\mathcal {X}}_A) \cdot \theta (x,\pi ,\sigma ) \end{aligned}$$

for all $$i\in J(\pi ,\sigma )$$.

### Proof

We define the following modified capacities. For $i\in [n]$, let

$$\begin{aligned} \begin{aligned} {\bar{\ell }}_{i}&{:}{=}{\left\{ \begin{array}{ll}0& \text{ if } c_{i} - A_i^\top \pi \le \sigma \, ,\\ x_{i}& \text{ if } c_{i} - A_i^\top \pi > \sigma \, . \end{array}\right. }\\ {{\bar{u}}}_{i}&{:}{=}{\left\{ \begin{array}{ll} u_{i}& \text{ if } c_{i} - A_i^\top \pi \ge -\sigma \, ,\\ x_{i}& \text{ if } c_{i} - A_i^\top \pi < -\sigma \, . \end{array}\right. } \end{aligned} \end{aligned}$$

We now consider the optimization problem

12 $$\begin{aligned} \begin{aligned} \min \  &\left\langle c,x\right\rangle \\ Ax&=b\\ {\bar{\ell }}\le x&\le {{\bar{u}}} \, . \end{aligned} \end{aligned}$$

This problem is feasible since *x* is a feasible solution; let ${{\bar{x}}}$ be an optimal solution.

### Claim 6.1

$${\bar{x}}_i=x_i$$ for every $$i\in J(\pi ,\sigma )$$.

### Proof

Consider a generalized path-circuit decomposition $\bar{x}-x=\sum _{k=1}^h g^k$. Since $A({{\bar{x}}}-x)={\textbf{0}}$, any conformal circuit decomposition may contain only inner vectors: $Ag^k={\textbf{0}}$ for all $k\le h$. We claim that $\textrm{supp}(g^k)\cap J(\pi ,\sigma )=\emptyset$ for all $k\in [h]$; this implies the statement. For a contradiction, assume there exists a $j\in \textrm{supp}(g^k)\cap J(\pi ,\sigma )$ for some $k\in [h]$.

The optimality of ${{\bar{x}}}$ implies that $0\ge \left\langle c,g^k\right\rangle =\left\langle c-A^\top \pi ,g^k\right\rangle$, as $Ag^k={\textbf{0}}$. Also, as we argue next, the definition of ${\bar{\ell }}$ implies that $g^k_i\ge 0$ for all $i\in [n]$ with $c_i-A_i^\top \pi >\sigma$; for in this case, for every such index *i*, by definition, ${\bar{\ell }}_i=x_i$, and thus ${{\bar{x}}}_i-x_i\ge 0$, that is, $g^k_i\ge 0$. Similarly, $g^k_i\le 0$ for all $i\in [n]$ with $c_i-A_i^\top \pi <-\sigma$. Thus $(c_i-A_i^\top \pi )g^k_i\ge 0$ whenever $|c_i-A_i^\top \pi |>\sigma$.

Let $S\subseteq \textrm{supp}(g^k)$ denote the set of indices *i* with $(c_i-A_i^\top \pi )g^k_i<0$, and let $\alpha {:}{=}\Vert g^k_S\Vert _\infty$. By the above, $j\notin S$ , and $(c_i-A_i^\top \pi )g^k_i\ge -\sigma \alpha$ for all $i\in S$.

If $S=\emptyset$, then $g_i^k=0$ must hold for all $i\in [n]$ with $|c_i-A_i^\top \pi |>\sigma$, implying ${{\bar{x}}}_i=x_i$ for $i\in J(\pi ,\sigma )$, completing the proof. Assume that $S\ne \emptyset$. The definition of $\kappa (A)\le \kappa ({\mathcal {X}}_A)$ implies that $|g^k_j|\ge \alpha /\kappa ({\mathcal {X}}_A)$. Hence,

$$\begin{aligned} \begin{aligned} 0&\ge \left\langle c,g^k\right\rangle =\left\langle c-A^\top \pi ,g^k\right\rangle \\&=\sum _{i\in \textrm{supp}(g^k)\setminus S}(c_i-A_i^\top \pi )g^k_i+\sum _{i\in S}(c_i-A_i^\top \pi )g^k_i\\&\ge (c_j-A_j^\top \pi )g^k_j-(n-1)\sigma \alpha \\&\ge n\cdot \kappa ({\mathcal {X}}_A)\cdot \sigma \cdot \frac{\alpha }{\kappa ({\mathcal {X}}_A)}-(n-1)\sigma \alpha >0\, , \end{aligned} \end{aligned}$$

a contradiction. $$\square $$

The following claim completes the proof of Lemma 4.3.

### Claim 6.2

Consider an optimal solution $x^*$ to LP(*A*, *b*, *c*, *u*) with $\Vert {{\bar{x}}}-x^*\Vert _1$ minimal. Then, $\Vert {{\bar{x}}}-x^*\Vert _\infty \le \kappa ({\mathcal {X}}_A)\cdot \theta (x,\pi ,\sigma )$.

### Proof

Consider a generalized path-circuit decomposition $x^*-\bar{x}=\sum _{k=1}^h g^k$. This may only contain inner vectors, since $A(x^*-{\bar{x}})={\textbf{0}}$. By the choice of $x^*$, we must have $\left\langle c,g^k\right\rangle <0$ for every *k*. Hence, ${{\bar{x}}}+\lambda g^k$ is not feasible to (12) for any $\lambda >0$, as otherwise we get a contradiction to the optimality of ${{\bar{x}}}$ to (12).

Therefore, for each *k*, there exists an $i_k\in \textrm{supp}(g^k)$ with ${\bar{\ell }}_{i_k}={{\bar{x}}}_{i_k}>0$ or ${{\bar{u}}}_{i_k}=\bar{x}_{i_k}<u_{i_k}$. By construction, ${\bar{x}}_{i_k} = x_{i_k}$ for these *i*. Using the definition of $\kappa (A)\le \kappa ({\mathcal {X}}_A)$ and the comformity of the decomposition, we get

$$\begin{aligned} \Vert {\bar{x}}-x^*\Vert _\infty \le \max _j \sum _{k=1}^h |g_j^k|&\le \sum _{k=1}^h\kappa (A)\cdot |g_{i_k}^k| \\&\le \kappa (A) \left[ \sum _{c_i -A_i^\top \pi \ge \sigma } x_i + \sum _{c_i -A_i^\top \pi < -\sigma }(u_i-x_i)\right] \\&\le \kappa ({\mathcal {X}}_A)\cdot \theta (x,\pi ,\sigma ), \end{aligned}$$

which is the claimed bound. $\square$

The lemma by from combining the previous two claims. $\square$

The next lemma bounds the difference in the optimum value if the right hand side changes; we will use it in the proof of Theorem 4.4.

### Lemma 6.3

Let $A\in {\mathbb {R}}^{m\times n}$, $c,u\in {\mathbb {R}}^n$, and $b,{{\bar{b}}}\in {\mathbb {R}}^m$. If both LPs are feasible, then

$$\begin{aligned} \big |\Phi (A,b,c,u)-\Phi (A,{{\bar{b}}},c,u)\big |\le \kappa ({\mathcal {X}}_A)\cdot \Vert c\Vert _1\cdot \Vert b-{{\bar{b}}}\Vert _1\, . \end{aligned}$$

### Proof

Let *x* be an optimal solution to LP(*A*, *b*, *c*, *u*) and ${{\bar{x}}}$ an optimal solution to $\textrm{LP}(A,{{\bar{b}}},c,u)$ such that $\Vert x-{{\bar{x}}}\Vert _1$ is minimal, and consider a generalized path-circuit decomposition ${{\bar{x}}}-x=\sum _{k=1}^h g^k$. By the choice of *x* and ${{\bar{x}}}$, the decomposition contains no inner circuits. Thus, by Lemma 3.4, $\Vert x-{{\bar{x}}}\Vert _\infty \le \kappa ({\mathcal {X}}_A)\cdot \Vert b-{{\bar{b}}}\Vert _1$. Therefore,

$$\begin{aligned}&\big |\Phi (A,b,c,u)-\Phi (A,{{\bar{b}}},c,u)\big |\\&\quad =\big |\left\langle c,x-\bar{x}\right\rangle \big |\le \Vert c\Vert _1\cdot \Vert x-{{\bar{x}}}\Vert _\infty \le \kappa ({\mathcal {X}}_A)\cdot \Vert c\Vert _1 \cdot \Vert b-{{\bar{b}}}\Vert _1\, , \end{aligned}$$

proving the claim. $\square$

We now restate and prove Theorem 4.4.

### Theorem 4.4

For $A\in {\mathbb {R}}^{m\times n}$, $b\in {\mathbb {R}}^m$, and $c,u\in {\mathbb {R}}^n$ such that LP(*A*, *b*, *c*, *u*) is feasible, let $\sigma {:}{=}\Vert c\Vert _\infty /(4n\cdot \lceil \kappa ({\mathcal {X}}_A)\rceil )$, and let $x\in [{\textbf{0}},u]$ and $\pi \in {\mathbb {R}}^m$ be a pair of (not necessarily feasible) primal and dual solutions. Then, LP$(A_{\scriptscriptstyle {\!N}},{{\bar{b}}},c_{\scriptscriptstyle {\!N}}- A_{\scriptscriptstyle {\!N}}^\top \pi ,u_{\scriptscriptstyle {\!N}})$ is feasible and, in addition satisfies the following:

- feasibility condition:
  7 $$\begin{aligned} \Vert b-{{\bar{b}}}-A_{\scriptscriptstyle {\!J_2}}u_{\scriptscriptstyle {\!J_2}}\Vert _1\le \theta (x,\pi ,\sigma )\cdot \Vert A\Vert _1+\Vert Ax-b\Vert _1\, , \end{aligned}$$
- optimality condition:
  8 $$\begin{aligned}&\big |\Phi (A_{\scriptscriptstyle {\!N}},{{\bar{b}}},c_{\scriptscriptstyle {\!N}}- A_{\scriptscriptstyle {\!N}}^\top \pi ,u_{\scriptscriptstyle {\!N}}) + \left\langle {{\bar{b}}},\pi \right\rangle +\left\langle c_{\scriptscriptstyle {\!J_2}},u_{\scriptscriptstyle {\!J_2}}\right\rangle -\Phi (A,b,c,u)\big | \nonumber \\&\quad = \big |\Phi (A_{\scriptscriptstyle {\!N}},{{\bar{b}}},c_{\scriptscriptstyle {\!N}},u_{\scriptscriptstyle {\!N}})+\left\langle c_{\scriptscriptstyle {\!J_2}},u_{\scriptscriptstyle {\!J_2}}\right\rangle -\Phi (A,b,c,u)\big |\nonumber \\&\quad \le \kappa ({\mathcal {X}}_A)\cdot \Vert c\Vert _1\cdot \Vert Ax-b\Vert _1\nonumber \\&\quad \quad + |J_1\cup J_2|\cdot \kappa ({\mathcal {X}}_A)\cdot \Vert c\Vert _{1}\cdot \big (2 + \kappa ({\mathcal {X}}_A) \Vert A\Vert _1\big )\cdot \theta (x,\pi ,\sigma )\, , \end{aligned}$$
- cost reduction: $$\Vert c_N-A_N^\top \pi \Vert _\infty \le \Vert c\Vert _\infty /4$$.

### Proof

Feasibility is trivial, since $x_{\scriptscriptstyle {\!N}}$ is a feasible solution. By the definition of $\theta (x,\pi ,\sigma )$,

$$\begin{aligned} \begin{aligned} \Vert b-{{\bar{b}}}-A_{\scriptscriptstyle {\!J_2}}{u_{\scriptscriptstyle {\!J_2}}}\Vert _1&\le \Vert b-Ax\Vert _1+ \Vert Ax-A_{\scriptscriptstyle {\!N}}x_{\scriptscriptstyle {\!N}}-A_{\scriptscriptstyle {\!J_2}}{u_{\scriptscriptstyle {\!J_2}}}\Vert _1\\&={}\Vert b-Ax\Vert _1+\Vert A_{\scriptscriptstyle {\!J_1}}x_{\scriptscriptstyle {\!J_1}}-A_{\scriptscriptstyle {\!J_2}}(u_{\scriptscriptstyle {\!J_2}}-x_{\scriptscriptstyle {\!J_2}})\Vert _1\\&\le {} \theta (x,\pi ,\sigma )\cdot \Vert A\Vert _1+\Vert Ax-b\Vert _1\, , \end{aligned} \end{aligned}$$

which finishes the proof of the feasibility condition.

To show the optimality condition, we will apply Lemmas 4.3 and 6.3 as follows. Consider the linear program $\textrm{LP}(A,Ax,c,u)$; *x* is a feasible solution. Let $x^*$ be an optimal solution to this LP as in Lemma 4.3. Let us define $y^*:==x^*|_{\scriptscriptstyle {\!N}}$ and $b^*:==A_{\scriptscriptstyle {\!N}}y^*$. Thus, $y^*$ is an optimal solution to $\textrm{LP}(A_{\scriptscriptstyle {\!N}},b^*,c_{\scriptscriptstyle {\!N}},u_{\scriptscriptstyle {\!N}})$.

For convenience, let $\rho :==2\cdot \kappa ({\mathcal {X}}_A)\cdot \theta (x,\pi ,\sigma )$, $J:==J_1\cup J_2$, and $k:==|J|$. For each $i\in J_2$, we have that $x^*_i\le x_i + \kappa ({\mathcal {X}}_A)\cdot \theta (x,\pi ,\sigma )\le \rho$, where the first inequality follows from Lemma 4.3 and the second one by the definition of $\theta (x,\pi ,\sigma )$. Similarly, for each $i\in J_1$, we obtain $x^*_i\ge u_i -\rho$. Recalling that $\Phi (A,Ax,c,u)=\left\langle c,x^*\right\rangle$, this yields the bound

13 $$\begin{aligned}&\big |\Phi (A_{\scriptscriptstyle {\!N}},b^*,c_{\scriptscriptstyle {\!N}},u_{\scriptscriptstyle {\!N}})+\left\langle c_{\scriptscriptstyle {\!J_2}},u_{\scriptscriptstyle {\!J_2}}\right\rangle -\Phi (A,Ax,c,u)\big |\nonumber \\&\quad = \big |\left\langle c_{\scriptscriptstyle {\!N}},y^*\right\rangle +\left\langle c_{\scriptscriptstyle {\!J_2}},u_{\scriptscriptstyle {\!J_2}}\right\rangle -\left\langle c,x^*\right\rangle \big |\le k\rho \Vert c\Vert _\infty \, . \end{aligned}$$

To finish the proof we need to bound the differences $|\Phi (A_{\scriptscriptstyle {\!N}},{{\bar{b}}},c_{\scriptscriptstyle {\!N}},u_N)-\Phi (A,Ax,c,u)|$, which we will do by applying Lemma 6.3. This yields

14 $$\begin{aligned} \big |\Phi (A,b,c,u)-\Phi (A,Ax,c,u)\big |\le \kappa ({\mathcal {X}}_A)\cdot \Vert c\Vert _1 \cdot \Vert Ax-b\Vert _1 \end{aligned}$$

and

15 $$\begin{aligned}&\big |\Phi (A_{\scriptscriptstyle {\!N}},{{\bar{b}}},c_{\scriptscriptstyle {\!N}},u_{\scriptscriptstyle {\!N}})-\Phi (A_{\scriptscriptstyle {\!N}},b^*,c_{\scriptscriptstyle {\!N}},u_{\scriptscriptstyle {\!N}})\big |\le \kappa ({\mathcal {X}}_A)\cdot \Vert c\Vert _1\cdot \Vert {{\bar{b}}}-b^*\Vert _1. \end{aligned}$$

We further bound $\Vert {{\bar{b}}}-b^*\Vert _1$ as follows:

16 $$\begin{aligned} \Vert {{\bar{b}}}-b^*\Vert _1&= \Vert A_{\scriptscriptstyle {\!N}}x_{\scriptscriptstyle {\!N}}- A_{\scriptscriptstyle {\!N}}y^*\Vert _1 \nonumber \\&= \Vert Ax - Ax^* - A_{\scriptscriptstyle {\!J}}(x_{\scriptscriptstyle {\!J}}-x_{\scriptscriptstyle {\!J}}^*)\Vert _1 \nonumber \\&= \Vert A_{\scriptscriptstyle {\!J}}(x_{\scriptscriptstyle {\!J}}-x_{\scriptscriptstyle {\!J}}^*)\Vert _1 \nonumber \\&\le k\cdot \Vert A\Vert _1 \cdot \kappa ({\mathcal {X}}_A) \cdot \theta (x,\pi ,\sigma ), \end{aligned}$$

where the inequality in the last line follows by how we obtained $x^*$ from Lemma 4.3. Finally, we conclude by combining (13), (14), (15), and (16):

$$\begin{aligned}&\left| \Phi (A_{\scriptscriptstyle {\!N}},{{\bar{b}}},c_{\scriptscriptstyle {\!N}},u_{\scriptscriptstyle {\!N}})+\left\langle c_{\scriptscriptstyle {\!J_2}},u_{\scriptscriptstyle {\!J_2}}\right\rangle -\Phi (A,b,c,u)\right| \\&\quad \le \left| \Phi (A_{\scriptscriptstyle {\!N}},b^*,c_{\scriptscriptstyle {\!N}},u_{\scriptscriptstyle {\!N}})+\left\langle c_{\scriptscriptstyle {\!J_2}},u_{\scriptscriptstyle {\!J_2}}\right\rangle -\Phi (A,Ax,c,u)\right| \\&\qquad + \big |\Phi (A,Ax,c,u) - \Phi (A,b,c,u)\big | \\&\qquad + \left| \Phi (A_{\scriptscriptstyle {\!N}},{{\bar{b}}},c_{\scriptscriptstyle {\!N}},u_{\scriptscriptstyle {\!N}})-\Phi (A_{\scriptscriptstyle {\!N}},b^*,c_{\scriptscriptstyle {\!N}},u_{\scriptscriptstyle {\!N}})\right| \\&\quad \le k\cdot \rho \cdot \Vert c\Vert _{\infty } + \kappa ({\mathcal {X}}_A)\cdot \Vert c\Vert _1 \cdot \Vert Ax-b\Vert _1 + \kappa ({\mathcal {X}}_A)\cdot \Vert c\Vert _1\cdot \Vert {{\bar{b}}}-b^*\Vert _1\\&\quad \le k\cdot \rho \cdot \Vert c\Vert _{\infty } + \kappa ({\mathcal {X}}_A)\cdot \Vert c\Vert _1 \cdot \Vert Ax-b\Vert _1\\&\qquad + k\cdot \kappa ({\mathcal {X}}_A)^2\cdot \Vert c\Vert _1\cdot \Vert A\Vert _1 \cdot \theta (x,\pi ,\sigma )\\&\quad = \kappa ({\mathcal {X}}_A)\cdot \Vert c\Vert _1 \cdot \Vert Ax-b\Vert _1 + \big [2k\cdot \kappa ({\mathcal {X}}_A)\cdot \Vert c\Vert _{\infty } \\&\qquad + k\cdot \kappa ({\mathcal {X}}_A)^2\cdot \Vert c\Vert _1\cdot \Vert A\Vert _1\big ] \cdot \theta (x,\pi ,\sigma )\\&\quad \le \kappa ({\mathcal {X}}_A)\cdot \Vert c\Vert _1 \cdot \Vert Ax-b\Vert _1 + k\cdot \kappa ({\mathcal {X}}_A)\cdot \Vert c\Vert _{1}\\&\qquad \cdot \big (2 + \kappa ({\mathcal {X}}_A)\cdot \Vert A\Vert _1\big ) \cdot \theta (x,\pi ,\sigma ). \end{aligned}$$

This proves the optimality condition. The third statement, that is, the cost reduction, directly follows from the definitions of $J_1$, $J_2$, *N* and $\sigma$. $\square$

## Analysis of the outer loop

We now prove the main statement for the outer loop.

### Theorem 5.1

If LP(*A*, *b*, *c*, *u*) is feasible, then Algorithm 1 returns a $\delta ^\textrm{feas}$-feasible solution that is $\delta ^\textrm{opt}$-optimal along with a $2 \delta ^\textrm{opt}$-certificate. It makes at most $\log _2(n\Vert u\Vert _1/\delta ^\textrm{opt})$ many recursive calls.

### Proof

We first show that the algorithm terminates with recursion depth at most $\log _2(n\Vert u\Vert _1/\delta ^\textrm{opt})$. By the cost reduction condition in Theorem 4.4, $\lambda \ge 2$ holds in each recursive call in Algorithm 1. If $\delta _0^\textrm{opt}$ denotes the initial $\delta ^\textrm{opt}$ and $\delta _j^\textrm{opt}$ denotes the $\delta ^\textrm{opt}$ of the *j*-th recursive call, then it follows that $\delta _j^\textrm{opt}\ge 2^j \delta _0^\textrm{opt}/n$. Thus, when $j\ge \log _2(n\Vert u\Vert _1/\delta _0^\textrm{opt})$, we have $\delta _j^\textrm{opt}\ge \Vert u\Vert _1$ and the algorithm terminates.

Now we prove the correctness of the algorithm by induction on the recursion depth. For the induction start, we assume that $\delta ^\textrm{opt}\ge \Vert u\Vert _1$, in which case an arbitrary $\delta ^\textrm{feas}$-feasible solution *x* is returned. Let $x^*$ be an optimal solution to LP(*A*, *b*, *c*, *u*). Since ${\textbf{0}}\le x,x^*\le u$, it follows that $\left\langle c,x\right\rangle -\Phi (A,b,c,u) = \left\langle c,x-x^*\right\rangle \le \Vert u\Vert _1\Vert c\Vert _\infty \le \delta ^\textrm{opt}\Vert c\Vert _\infty$, proving $\delta ^\textrm{opt}$-optimality.

For the inductive step, assume $\delta ^\textrm{opt}< \Vert u\Vert _1$. Then the algorithm invokes the subroutine GetPrimalDualPair followed by a recursive call to itself. Recall that the subroutine GetPrimalDualPair returns $(x,\pi )$ that satisfy the three bounds stated as part of the subroutine. We distinguish two cases depending on whether $J_1\cup J_2$ is empty or not.

If $J_1\cup J_2=\emptyset$, then the induction hypothesis applied to the call in line 10 implies that $x^\textrm{out}$ is $\delta ^\textrm{feas}$-feasible for the original system. Moreover, it is a $\lambda \delta ^\textrm{opt}$-optimal solution to $\textrm{LP}(A,b,c^\textrm{new},u)$, with $\lambda = \Vert c\Vert _\infty /(2\Vert c^\textrm{new}\Vert _\infty )$ in this case. Note that $\Phi (A,b,c^\textrm{new},u)=\Phi (A,b,c,u)-\left\langle \pi ,b\right\rangle$. This implies:

$$\begin{aligned}&|\left\langle c,x^\textrm{out}\right\rangle - \Phi (A,b,c,u)|\le |\left\langle c-c^\textrm{new},x^\textrm{out}\right\rangle -\left\langle \pi ,b\right\rangle |\\&\qquad +|\left\langle c^\textrm{new},x^\textrm{out}\right\rangle -\Phi (A,b,c^\textrm{new},u)|\\&\quad \le |\left\langle \pi ,Ax^\textrm{out}-b\right\rangle |+\lambda \delta ^\textrm{opt}\Vert c^\textrm{new}\Vert _\infty {~~~~\text {(as }\, x^\textrm{out}\,\text { is}\, \lambda \delta ^\textrm{opt}-\text {optimal)}}\\&\quad \le \Vert \pi \Vert _{\infty } \cdot \delta ^\textrm{feas}\Vert A\Vert _1+\lambda \delta ^\textrm{opt}\Vert c^\textrm{new}\Vert _\infty ~~~~\text {(as}\, x^\textrm{out}\,\text {is}\, \delta ^\textrm{feas}-\text {feasible)}\\&\quad \le \frac{\delta ^\textrm{opt}\Vert c\Vert _\infty }{2\delta ^\textrm{feas}\Vert A\Vert _1} \cdot \delta ^\textrm{feas}\Vert A\Vert _1+\lambda \delta ^\textrm{opt}\Vert c^\textrm{new}\Vert _\infty \\&\qquad \,\,{\text {(using the upper bound on}}\,\, \Vert \pi \Vert _{\infty } \,{\text { in }}\, \texttt {GetPrimalDualPair}{}\\&\qquad \,{\text {and the lower bound on }}\, \delta ^\textrm{opt}/\delta ^\textrm{feas}\,{\text {in }} \,\texttt {SolveLP}\text {)}\\&\quad \le \frac{\delta ^\textrm{opt}}{2}\Vert c\Vert _\infty +\frac{\delta ^\textrm{opt}}{2}\Vert c\Vert _\infty = \delta ^\textrm{opt}\Vert c\Vert _\infty , \end{aligned}$$

finishing the first case.

Otherwise, if $J_1\cup J_2\ne \emptyset$, note that $\textrm{LP}(A_{\scriptscriptstyle {\!N}},{{\bar{b}}}, c_{\scriptscriptstyle {\!N}}^\textrm{new}, u_{\scriptscriptstyle {\!N}})$ is feasible because $x_{\scriptscriptstyle {\!N}}$ is a feasible solution. Thus, we can apply the induction hypothesis to the recursive call, and obtain that $x_{\scriptscriptstyle {\!N}}^\textrm{out}$ is $(\delta ^\textrm{feas}\cdot |N|/n)$-feasible and $(\lambda \delta ^\textrm{opt}\cdot |N|/n)$-optimal for $\textrm{LP}(A_{\scriptscriptstyle {\!N}},{{\bar{b}}}, c_{\scriptscriptstyle {\!N}}^\textrm{new}, u_{\scriptscriptstyle {\!N}})$. We combine this with Theorem 4.4 and the properties of the subroutine to prove first the $\delta ^\textrm{feas}$-feasiblity and then the $\delta ^\textrm{opt}$-optimality of $x^\textrm{out}$ for the original system.

With respect to feasiblity, we obtain:

$$\begin{aligned} \Vert A x^\textrm{out}- b \Vert _1&\le \Vert A_{\scriptscriptstyle {\!N}}x_{\scriptscriptstyle {\!N}}^\textrm{out}- {{\bar{b}}}\Vert _1 + \Vert {{\bar{b}}}+A_{\scriptscriptstyle {\!J_2}}u_{\scriptscriptstyle {\!J_2}}-b\Vert _1~~~~\text {(as}\, A x^\textrm{out}= A_{\scriptscriptstyle {\!N}}x_{\scriptscriptstyle {\!N}}^\textrm{out}+ A_{\scriptscriptstyle {\!J_2}}u_{\scriptscriptstyle {\!J_2}}\text {)}\\&\le (\delta ^\textrm{feas}\cdot |N|/n)\Vert A_{\scriptscriptstyle {\!N}}\Vert _1 +\theta (x,\pi ,\sigma )\cdot \Vert A\Vert _1\\&\quad + \Vert Ax-b\Vert _1~~~~\text {(explanation below)}\\&\le (\delta ^\textrm{feas}\cdot |N|/n)\Vert A_{\scriptscriptstyle {\!N}}\Vert _1 + \delta ^\textrm{feas}\Vert A\Vert _1/n~~~~~~~~~~~~~~~~\text {(explanation below)}\\&\le \delta ^\textrm{feas}\Vert A\Vert _1. \end{aligned}$$

The second inequality uses induction for the first term and Theorem 4.4 for the second term. The third inequality follows because GetPrimalDualPair returns a solution bounding the second and third terms in the second line, the RHS of (7), by the second term in the third line.

To prove approximate optimality, we first focus on the coordinates in *N* and obtain the following bound. (See below for explanations.)

17 $$\begin{aligned} |\left\langle c_{\scriptscriptstyle {\!N}},x_{\scriptscriptstyle {\!N}}^\textrm{out}\right\rangle -&\Phi (A_{\scriptscriptstyle {\!N}},{{\bar{b}}},c_{\scriptscriptstyle {\!N}}^\textrm{new},u_{\scriptscriptstyle {\!N}}) - \left\langle \pi ,{{\bar{b}}}\right\rangle |\nonumber \\&\le |\left\langle c_{\scriptscriptstyle {\!N}}-c_{\scriptscriptstyle {\!N}}^\textrm{new},x_{\scriptscriptstyle {\!N}}^\textrm{out}\right\rangle -\left\langle \pi ,{{\bar{b}}}\right\rangle |+|\left\langle c_{\scriptscriptstyle {\!N}}^\textrm{new},x_{\scriptscriptstyle {\!N}}^\textrm{out}\right\rangle -\Phi (A_{\scriptscriptstyle {\!N}},{{\bar{b}}},c_{\scriptscriptstyle {\!N}}^\textrm{new},u_{\scriptscriptstyle {\!N}})|\nonumber \\&\le |\left\langle \pi ,A_{\scriptscriptstyle {\!N}}x_{\scriptscriptstyle {\!N}}^\textrm{out}-{{\bar{b}}}\right\rangle |+(\lambda \delta ^\textrm{opt}\cdot |N|/n)\cdot |c^\textrm{new}\Vert _\infty \nonumber \\&\le \Vert \pi \Vert _\infty \Vert A_{\scriptscriptstyle {\!N}}\Vert _1 \delta ^\textrm{feas}\cdot \frac{|N|}{n}+ \frac{\delta ^\textrm{opt}|N |}{2n}\cdot \Vert c\Vert _\infty \nonumber \\&\le \frac{\delta ^\textrm{opt}\Vert c\Vert _\infty }{2\delta ^\textrm{feas}\Vert A\Vert _1} \Vert A_{\scriptscriptstyle {\!N}}\Vert _1 \delta ^\textrm{feas}\cdot \frac{|N|}{n} + \frac{\delta ^\textrm{opt}|N |}{2n}\Vert c\Vert _\infty \nonumber \\&\le (\delta ^\textrm{opt}\cdot |N|/n)\Vert c\Vert _\infty . \end{aligned}$$

The bound $\Vert \pi \Vert _\infty \Vert A_{\scriptscriptstyle {\!N}}\Vert _1 \delta ^\textrm{feas}\cdot |N|/n$ on the first term in the third line follows as $x_{\scriptscriptstyle {\!N}}^\textrm{out}$ is a $\delta ^\textrm{feas}|N| / n$ feasible solution. In turn, the bound on this term follows because of the bound on $\Vert \pi \Vert _\infty$ in GetPrimalDualPair and $0<\delta ^\textrm{feas}(8 n \sqrt{m} \cdot \kappa ({\mathcal {X}}_A) \Vert A\Vert _1 )\le \delta ^\textrm{opt}\le \Vert u\Vert _1$ (the second inequality is an input constraint for SolveLP, and the final inequality arises due to computing *N* in SolveLP only when $\delta ^\textrm{opt}<\Vert u\Vert _1$). Finally,

$$\begin{aligned}&|\left\langle c,x^\textrm{out}\right\rangle -\Phi (A,b,c,u)|\le |\left\langle c_{\scriptscriptstyle {\!N}},x_{\scriptscriptstyle {\!N}}^\textrm{out}\right\rangle - \Phi (A_{\scriptscriptstyle {\!N}},{{\bar{b}}},c_{\scriptscriptstyle {\!N}}^\textrm{new},u_{\scriptscriptstyle {\!N}}) - \left\langle \pi ,{{\bar{b}}}\right\rangle |\\&\qquad + |\Phi (A_{\scriptscriptstyle {\!N}},{{\bar{b}}},c_{\scriptscriptstyle {\!N}}^\textrm{new},u_{\scriptscriptstyle {\!N}}) + \left\langle \pi ,{{\bar{b}}}\right\rangle +\left\langle c_{\scriptscriptstyle {\!J_2}},u_{\scriptscriptstyle {\!J_2}}\right\rangle -\Phi (A,b,c,u)|\\&\quad \le (\delta ^\textrm{opt}\cdot |N|/n)\Vert c\Vert _\infty + \delta ^\textrm{opt}\Vert c\Vert _\infty /n\\&\quad \le \delta ^\textrm{opt}\Vert c\Vert _\infty . \end{aligned}$$

To obtain the first inequality, recall that $x^\textrm{out}$ is a combination of $x_{\scriptscriptstyle {\!N}}^\textrm{out}$, $x_{\scriptscriptstyle {\!J_1}}^\textrm{out}=0$, and $x_{\scriptscriptstyle {\!J_1}}^\textrm{out}=u_{\scriptscriptstyle {\!J_2}}$. For the next inequality, note that the first term on line 2 is bounded by $(\delta ^\textrm{opt}\cdot |N|/n)\Vert c\Vert _\infty$ using (17), and the second term is bounded by the LHS of (8), which is bounded by $\delta ^\textrm{opt}\Vert c\Vert _\infty /n$ according to the second condition of GetPrimalDualPair. This concludes the second case. $\square$

## Analysis of the inner loop

In this section, we prove Theorem 5.4 on the correctness and running time of the inner loop. The proof uses three main lemmas. The first one bounds the number of iterations in R-FGM, using Theorem 2.5. This lemma will be proved in Sect. 8.2. Recall that the approximation factor $\varepsilon$ was chosen to be $\varepsilon = 1 /(8n\cdot \lceil \kappa ({\mathcal {X}}_{A})\rceil )$.

### Lemma 8.1

After $O\left( k \sqrt{n} m^2 \Vert A\Vert _1^2 \cdot {\bar{\kappa }}^2({\mathcal {X}}_{A}) / \varepsilon \right)$ iterations, R-FGM returns an $\textrm{e}^{-2k} \Vert b \Vert _2^2 / (2 \Vert A\Vert _1^2)$-approximate minimizer of $F_{\tau }$.

Recall that $F_{\tau }^\star = \min \{F_{\tau }(x)\mid x\in [{\textbf{0}},u]\}$ denotes the optimum value of $F_{\tau }$ defined in (10). The next lemma strengthens Proposition 5.2, by asserting a Lipschitz property of $F_{\tau }^\star$ as a function of $\tau$.

### Lemma 8.2

$F_{\tau }^\star$ is a non-increasing and continuous function of $\tau$. In addition, If $F_{\tau }^\star \le \overline{{\mathcal {C}}}^2 \zeta$, then $F_{\tau - \overline{{\mathcal {C}}}\sqrt{\zeta }/2}^\star \le (2\overline{{\mathcal {C}}}^2 - 1) \zeta$.

### Proof

The first part is simple and was already stated as Proposition 5.2. Let $x^*$ be the optimal solution of $F_{\tau }^\star$. Let

$$\begin{aligned} \alpha {:}{=}\max \left\{ 0, \left\langle {\hat{c}}, x^* \right\rangle - \tau \right\} \,\quad \text{ and }\quad \Delta {:}{=} \overline{{\mathcal {C}}}\sqrt{\zeta }/2 \, .\end{aligned}$$

The lemma follows by showing that

18 $$\begin{aligned} F_{ \tau - \Delta }(x^*)-F_{\tau }^\star \le (\overline{{\mathcal {C}}}^2 - 1) \zeta . \end{aligned}$$

Since $F_{\tau }^\star \le \overline{{\mathcal {C}}}^2 \zeta$ and $F_{\tau }^\star \ge \frac{1}{2}\alpha ^2$, we have $\alpha \le \overline{{\mathcal {C}}}\sqrt{2 \zeta } = 2 \sqrt{2} \Delta$. Therefore,

$$\begin{aligned} F_{ \tau - \Delta }(x^*) - F_{\tau }^\star&=\frac{1}{2} (\max \{0, \left\langle {\hat{c}}, x^* \right\rangle - \tau + \Delta \} )^2 - \frac{1}{2}(\max \{0, \langle {\hat{c}}, x^* \rangle - \tau \} )^2 \\&\le \frac{1}{2}(\alpha +\Delta )^2 -\frac{1}{2}\alpha ^2\\&= \alpha \Delta +\frac{1}{2}\Delta ^2\le \left( 2\sqrt{2}+\frac{1}{2}\right) \Delta ^2\, . \end{aligned}$$

Using the above bound, we get (18) from

$$\begin{aligned}&F_{ \tau - \Delta }(x^*) - F_{\tau }^\star \le \left( 2\sqrt{2}+\frac{1}{2}\right) \Delta ^2 = \left( \frac{1}{\sqrt{2}} + \frac{1}{8}\right) \overline{{\mathcal {C}}}^2 \zeta \le (\overline{{\mathcal {C}}}^2 - 1)\zeta \, . \end{aligned}$$

The last inequality follows by Lemma 5.3. $\square$

The third lemma shows that if $F_{\tau }(x)$ lies in the appropriate interval, then the output satisfies the desired properties of the algorithm. The proof is given in Sect. 8.1.

### Lemma 8.3

Let *x* be a $\zeta$-approximate minimizer and $\pi$ as defined in the algorithm. Assume that $F_{\tau }(x) \in [\overline{{\mathcal {C}}}^2 \zeta , 2 \overline{{\mathcal {C}}}^2 \zeta ]$. Then

(i) $$ \theta (x, \pi , \sigma ) \le n \sqrt{\zeta } / 2$$,
(ii) $$ \Vert Ax - b\Vert _1 \le 128 n^2 m \cdot \kappa ^2({\mathcal {X}}_A) \cdot {\Vert A\Vert _1^2} \sqrt{\zeta }$$, and
(iii) $$\Vert \pi \Vert _{\infty } \le 4n \sqrt{m} \cdot \kappa ({\mathcal {X}}_A)\cdot \Vert c\Vert _{\infty } $$.

We now give the proof of Theorem 5.4 using these three lemmas.

### Theorem 5.4

Assume LP(*A*, *b*, *c*, *u*) is feasible. Algorithm 2 makes $O\big (\log \big [\Vert u\Vert _1 nm\cdot \kappa ({\mathcal {X}}_{A})/\delta ^\textrm{feas}\big ]\big )$ calls to R-FGM, and altogether these calls use $O\big ( n^{1.5} m^2 \Vert A\Vert ^2_1 \cdot {\bar{\kappa }}^3({\mathcal {X}}_{A}) \log ^2 \big [\Vert u\Vert _1 n m \cdot \Vert A\Vert _1 \kappa ({\mathcal {X}}_A)/\delta ^\textrm{feas}\big ]\big )$ iterations. On terminating, it outputs $(x, \pi )$ satisfying:

(i) $$\theta (x, \pi , \sigma ) \cdot \Vert A\Vert _1 + \Vert Ax - b\Vert _1 \le \delta ^\textrm{feas}\Vert A\Vert _1 / n$$.
(ii) $$\kappa ({\mathcal {X}}_A)\cdot \Vert c\Vert _1\cdot \Vert Ax-b\Vert _1 + |J_1\cup J_2|\cdot \kappa ({\mathcal {X}}_A)\cdot \Vert c\Vert _{1}\cdot \big (2 + \kappa ({\mathcal {X}}_A) \Vert A\Vert _1\big )\cdot \theta (x,\pi ,\sigma ) \le \delta ^\textrm{opt}\Vert c\Vert _\infty / n $$.
(iii) $$\Vert \pi \Vert _{\infty } \le 4n \sqrt{m} \cdot \kappa ({\mathcal {X}}_A)\cdot \Vert c\Vert _{\infty } $$.

### Proof

First of all, we need to show that the algorithm eventually outputs a primal dual pair $(x, \pi )$, i.e., the condition $F_{\tau }(x) \in [\overline{{\mathcal {C}}}^2 \zeta , 2 \overline{{\mathcal {C}}}^2 \zeta ]$ will be met, and the claimed bound on the number of calls to R-FGM is applicable. This follows by the next claim.

### Claim 8.4

$$\tau ^{+} - \tau ^{-} \ge \overline{{\mathcal {C}}}\sqrt{ \zeta } / 2$$ throughout Algorithm 2.

### Proof

We show the following invariant property of the algorithm:

$$F^*_{\tau ^-} > (2 \overline{{\mathcal {C}}}^2 - 1) \zeta \,\text {and}\, F^*_{ \tau ^+} < \overline{{\mathcal {C}}}^2 \zeta .$$

Initially, for any feasible *x* to LP(*A*, *b*, *c*, *u*), we have $F_{\tau ^+}(x) =0$, and therefore $F^*_{ \tau ^+} = 0$. Also, $F^*_{\tau ^-} \ge 2 \overline{{\mathcal {C}}}^2 \zeta$ as $F_{\tau ^{-}} \ge \frac{1}{2}(\max \{0, \langle {\hat{c}}, x \rangle - \tau ^{-}\})^2 \ge \frac{1}{2 }( 2 \overline{{\mathcal {C}}}\sqrt{ \zeta })^2 = 2 \overline{{\mathcal {C}}}^2 \zeta$, noting that $\left\langle {\hat{c}},x\right\rangle \ge -\Vert {\hat{c}}\Vert _\infty \Vert u\Vert _1\ge -\Vert u_1\Vert$. During the algorithm, each computed *x* is a $\zeta$-approximate minimizer of $F_{\tau }$, and therefore the updating of $\tau ^-$ and $\tau ^+$ in steps 5–8 maintains the invariant.

For a contradiction, assume $\tau ^{+} - \tau ^{-} \le \overline{{\mathcal {C}}}\sqrt{ \zeta } / 2$ at some point. Then, $F^*_{\tau ^+} < \overline{{\mathcal {C}}}^2 \zeta$ and Lemma 8.2 imply $F^*_{ \tau ^-} \le (2 \overline{{\mathcal {C}}}^2 - 1) \zeta$, a contradiction. $\square$

To bound the number of calls to R-FGM, note that initially $\tau ^+-\tau ^-=2\Vert u\Vert _1+2\overline{{\mathcal {C}}}\sqrt{ \zeta } \le 4 \Vert u\Vert _1$ as $\Vert u\Vert _1 \ge {\delta ^\textrm{feas}}$, as required for the input of $\texttt {GetPrimalDualPair}$; and $\tau ^+-\tau ^-$ is halved in every iteration. According to the above claim, it never goes below $\overline{{\mathcal {C}}}\sqrt{ \zeta } / 2$, and applying Lemma 5.3 yields the following bound on the number of calls:

$$\begin{aligned} O\left( \log \left( {\Vert u\Vert _1}/{(\overline{{\mathcal {C}}}\sqrt{ \zeta })}\right) \right) =O\left( \log \left( \Vert u\Vert _1 nm\cdot \kappa ({\mathcal {X}}_{A})/\delta ^\textrm{feas}\right) \right) \, . \end{aligned}$$

In each R-FGM call, by Lemma 8.1, to obtain a $\zeta$-approximate minimizer takes at most $O(k {\sqrt{n} m^2} \Vert A\Vert _1^2\cdot {\bar{\kappa }}^2({\mathcal {X}}_{A}) / \varepsilon )$ steps, where $k = O\left( \log (\Vert b\Vert _2^2/(\Vert A\Vert _1^2 \zeta )\right)$ and $\varepsilon = 1 / [8n \cdot \kappa ({\mathcal {X}}_{A})]$. The claimed bound follows as $\Vert b\Vert _2^2 \le \Vert b\Vert _1^2 \le \Vert A\Vert ^2_1 \cdot \Vert u\Vert ^2_1$, which follows from the feasibility assumption.

The bounds on *x* and $\pi$ asserted in the theorem follow by simple calculation from the bounds in Lemma 8.3 and the definition of $\zeta$ in (11). $\square$

### Optimality bounds from the potential function

Recall the parameters $\varepsilon = 1 /(8n\cdot \lceil \kappa ({\mathcal {X}}_{A})\rceil )$, $\sigma =\Vert c\Vert _\infty /(4n\cdot \lceil \kappa ({\mathcal {X}}_A)\rceil )=2\varepsilon \Vert c\Vert _\infty$. Recall that in Algorithm 2, we defined

$$\begin{aligned} \alpha (x){:}{=} \alpha = \max \{0, \langle {\hat{c}}, x \rangle - \tau \}\, ,\quad \text{ and }\quad \pi (x) {:}{=} \pi = \frac{ \Vert c\Vert _{\infty }}{\Vert A\Vert ^2_1\alpha (x) }(b-Ax)\, \end{aligned}$$

Let us further define

19 $$\begin{aligned} \beta (x){:}{=}\frac{\Vert Ax-b\Vert _2}{\Vert A\Vert _1}\, . \end{aligned}$$

With this notation, we can write

$$\begin{aligned} F_{\tau }(x) = \frac{1}{2}\alpha (x)^2 + \frac{1}{2}\beta (x)^2\, . \end{aligned}$$

We will also often use the gradient, which can be expressed as

20 $$\begin{aligned} \nabla F_{\tau }(x)=\alpha (x)\left( {\hat{c}}-\frac{A^\top \pi (x)}{\Vert c\Vert _\infty }\right) \, . \end{aligned}$$

When *x* is clear from the context, we simply write $\alpha$, $\beta$, and $\pi$. We will use a simple convexity statement, formulated in the following general form.

#### Proposition 8.5

Let $f\, :\, {\mathbb {R}}^n\rightarrow {\mathbb {R}}$ be a continuously differentiable convex function with $[{\textbf{0}},u]\subseteq \textrm{dom}(f)$, such that for some *M*, *f* satisfies the following smoothness property: for every index $i\in [n]$, for every pair $x,y\in {\mathbb {R}}^n$ such that $x_j=y_j$ for all $j\ne i$,

21 $$\begin{aligned} |\nabla _i f(x)-\nabla _i f(y)| \le M|x_i-y_i|\, . \end{aligned}$$

Also, suppose that for some $\eta >0$, *x* is an $\eta$-approximate minimizer to the program

$$\begin{aligned} \min f(x)\quad \text{ s.t. } \quad x\in [{\textbf{0}},u]\, . \end{aligned}$$

Then, for any $i\in [n]$, the following hold:

(i) If $$\nabla _i f(x)\ge 2\sqrt{M\eta }$$, then $$x_i\le \sqrt{\eta / M}$$.
(ii) If $\nabla _i f(x)\le -2\sqrt{M\eta }$, then $x_i\ge u_i-\sqrt{\eta / M}$.

#### Proof

We only prove part (i); part (ii) follows analogously. For a contradiction, assume that for some $i\in [n]$, $\nabla _i f(x)\ge 2\sqrt{M \eta }$ and $x_i>\sqrt{\eta / M}$. Let us define $z\in {\mathbb {R}}^n$ with $z_i{:}{=}x_i-\sqrt{\eta / M}>0$ and $z_j{:}{=}x_j$ for $j\ne i$. Thus, $z\in [{\textbf{0}},u]$, and by the smoothness property, $\nabla _i f(z)\ge \nabla _i f(x)-\sqrt{M\eta }\ge \sqrt{M\eta }$. By convexity,

$$\begin{aligned} f(x)&\ge f(z)+\left\langle \nabla f(z),x-z\right\rangle = f(z)+\nabla _i f(z)\cdot \sqrt{\eta / M}\ge f(z)\\&\quad +\sqrt{M \eta }\cdot \sqrt{\eta / M}>f(z)+\eta \, , \end{aligned}$$

using $M\ge 1$. This is a contradiction to the assumption that *x* is an $\eta$-approximate minimizer. $\square$

The next lemma is used to prove the first key ingredient of Lemma 8.3, an upper bound on $\theta (x, \pi , \sigma )$, provided that $\alpha (x)$ is sufficiently large.

#### Lemma 8.6

Suppose *x* is a $\zeta$-approximate minimizer of $F_{\tau }(x)$ satisfying $\varepsilon \cdot \alpha (x) \ge 4 \sqrt{\zeta }$, and $\pi =\pi (x)$. Then $\theta (x, \pi , \sigma ) \le n\sqrt{ \zeta } / 2$.

#### Proof

We use $\alpha =\alpha (x)$, $\beta =\beta (x)$, $\pi =\pi (x)$ throughout. Recall that $\theta (x, \pi , \sigma ) = \sum _{i : c_i - A_i^\top \pi > \sigma } x_i + \sum _{i: c_i - A_i^\top \pi < - \sigma } (u_i - x_i)$, and that the vector ${\hat{c}}$ was obtained by normalizing as $c/\Vert c\Vert _\infty$, and then rounding down each positive entry to the nearest integer multiple of $\varepsilon$, and rounding up each negative entry to the nearest integer multiple of $\varepsilon$. Also noting that $\sigma =2\varepsilon \Vert c\Vert _\infty$, we obtain the following upper bound in terms of ${\hat{c}}$.

22 $$\begin{aligned} \theta (x, \pi , \sigma ) \le \sum _{i : {\hat{c}}_i - A_i^\top \pi / \Vert c\Vert _{\infty } > \varepsilon } x_i + \sum _{i: {\hat{c}}_i - A_i^\top \pi / \Vert c\Vert _{\infty } < - \varepsilon } (u_i - x_i). \end{aligned}$$

We will show each of the terms $x_i$ and $u_i-x_i$ in these two sums is bounded by $\frac{\sqrt{\zeta }}{2}$, and then the result is immediate. Recall from (20) that

$$\begin{aligned} {\hat{c}}_i - A_i^\top \pi / \Vert c\Vert _{\infty }=\nabla _i F_{\tau }(x) / \alpha \, . \end{aligned}$$

We apply Proposition 8.5 to $F_{\tau }(x)$ and $\eta =\zeta$. From (20), a simple calculation shows that $f(x)=F_{\tau }(x)$ satisfies the smoothness bound (21) with $M=2$. Namely, for any $i\in [n]$ and for any $x,y\in {\mathbb {R}}^n$ such that $x_j=y_j$ for $i\ne j$,

$$\begin{aligned} |\nabla _i F_{\tau }(x) - \nabla _i F_{\tau }(y)|&\le \Vert {\hat{c}}\Vert _{\infty } \Vert x - y\Vert _1 + |A_i^\top A_i| \cdot \Vert (x - y)\Vert _{1} / \Vert A\Vert _1^2 \le 4 |x_i - y_i|\, . \end{aligned}$$

${\hat{c}}_i - A_i^\top \pi / \Vert c\Vert _{\infty } > \varepsilon$ is equivalent to $\nabla _i F_{\tau }(x)\ge \varepsilon \alpha$. By the assumption of the lemma, $\varepsilon \alpha \ge 4\sqrt{\zeta }=2\sqrt{M\zeta }$.

Thus, Proposition 8.5(i) implies that whenever ${\hat{c}}_i - A_i^\top \pi / \Vert c\Vert _{\infty } > \varepsilon$, we must have $x_i\le \sqrt{\zeta }/2$. Similarly, Proposition 8.5(ii) implies that whenever ${\hat{c}}_i - A_i^\top \pi / \Vert c\Vert _{\infty } < - \varepsilon$, we have $u_i-x_i\le \sqrt{\zeta }/2$. This completes the proof. $\square$

Note the previous lemma requires a lower bound on $\alpha$. Our second lemma shows that this requirement is satisfied if one get a good approximate minimizer with a sufficiently large function value.

#### Lemma 8.7

Suppose *x* is a $\zeta$-approximate minimizer of $F_{\tau }(x)$, satisfying $F_{\tau }(x) \ge 10 {\mathcal {C}}^2\zeta$. Then, $\alpha (x) \ge \frac{1}{2{\mathcal {C}}} \sqrt{F_{\tau }(x)}$.

The proof relies on the following statement:

#### Proposition 8.8

Assume LP(*A*, *b*, *c*, *u*) is feasible, and let $x\in [{\textbf{0}},u]$ with $\beta (x) > 0$. Then, for any $0 \le \mu \le 1$, there exists a solution $x'\in [{\textbf{0}},u]$ such that $\alpha (x') \le \alpha (x) + {\mu } {\mathcal {C}}\beta (x)$ and $\beta (x') \le (1 - \mu ) \beta (x)$.

#### Proof

Let $z\in {\mathbb {R}}^n$ be chosen as

$$\begin{aligned} z{:}{=}\arg \min \{\Vert z-x\Vert ^2\mid Az=b\, ,\, z\in [{\textbf{0}},u]\}\, \quad \text{ and } \text{ set }\quad x' {:}{=} \mu z + (1 - \mu ) x\, . \end{aligned}$$

Clearly, $\beta (x') = (1 - \mu ) \beta (x)$. By Lemma 3.4, $\Vert z - x\Vert _{\infty } \le \sqrt{m} \cdot \kappa ({{\mathcal {X}}_{A}})\cdot \Vert Ax - b\Vert _ 2$. Therefore, $\alpha$ increases by at most

$$\begin{aligned} \langle {\hat{c}}, (\mu z + (1 - \mu ) x) - x \rangle&\le \mu \cdot \Vert {\hat{c}}\Vert _1 \Vert z - x \Vert _{\infty } \le \mu n \cdot \sqrt{m} \cdot \kappa ({{\mathcal {X}}_{A}})\cdot \Vert Ax - b\Vert _ 2 \\&= \mu n \cdot \sqrt{m} \cdot \kappa ({{\mathcal {X}}_{A}})\cdot \Vert A\Vert _1 \beta (x) \le \mu {\mathcal {C}}\beta (x) \, , \end{aligned}$$

recalling the definition of ${\mathcal {C}}$ in (11). $\square$

#### Proof of Lemma 8.7

We distinguish two cases.

***Case i:*** $$2{\mathcal {C}}\alpha (x)\ge \beta (x)$$.

In this case,

$$\begin{aligned} F_{\tau }(x)&= \frac{1}{2} \alpha (x) ^2 + \frac{1}{2} \beta (x)^2 \le \frac{4{\mathcal {C}}^2+1}{2} \alpha (x)^2 \, . \end{aligned}$$

Thus, the claimed $\alpha (x) \ge \frac{1}{2{\mathcal {C}}} \sqrt{F_{\tau }(x)}$ follows by recalling ${\mathcal {C}}\ge 1$ (Lemma 5.3).

***Case ii:*** $$2{\mathcal {C}}\alpha (x)< \beta (x)$$.

Let us use Proposition 8.8 with

$$\begin{aligned} \mu {:}{=} \frac{\beta (x) - {\mathcal {C}}\alpha (x) }{({\mathcal {C}}^2 +1)\beta (x) }\, . \end{aligned}$$

Clearly, $\mu \in [0,1]$. Thus, there exists $x'\in [{\textbf{0}},u]$ such that $\alpha (x') \le \alpha (x) + \mu {\mathcal {C}}\beta (x)$ and $\beta (x') \le (1 - \mu ) \beta (x)$. Therefore,

$$\begin{aligned} F_{\tau }(x')&= \frac{1}{2} \alpha (x')^2 + \frac{1}{2} \beta (x')^2 \le \frac{1}{2} (\alpha (x)+ \mu {\mathcal {C}}\beta (x))^2 + \frac{1}{2} (1 - \mu )^2 \beta (x)^2. \end{aligned}$$

Above, we picked $\mu$ to minimize this expression, and some calculation yields

$$\begin{aligned} F_{\tau }(x') \le \frac{ (\alpha (x) + {\mathcal {C}}\beta (x))^2}{2 ({\mathcal {C}}^2 + 1)}.\, \end{aligned}$$

Further calculation yields

$$\begin{aligned} F_{\tau }(x) - F_{\tau }(x') \ge \frac{ ({\mathcal {C}}\alpha (x) - \beta (x))^2}{2 ({\mathcal {C}}^2 + 1)}> \frac{\beta (x)^2}{8 ({\mathcal {C}}^2 + 1)}, \end{aligned}$$

where the last inequality uses the assumption $2{\mathcal {C}}\alpha (x) < \beta (x)$. The same condition also implies that $F_{\tau }(x) = \frac{1}{2} \alpha (x)^2 + \frac{1}{2} \beta (x)^2 \le \frac{(4 {\mathcal {C}}^2 + 1)\beta (x)^2}{8 {\mathcal {C}}^2 }$. Using ${\mathcal {C}}\ge 1$, these two bounds in turn, and also the lower bound on $F_{\tau }(x)$ assumed in the lemma, we obtain

$$\begin{aligned} F_{\tau }(x) - F_{\tau }(x') > \frac{\beta ^2(x)}{8 ({\mathcal {C}}^2 + 1)} \ge \frac{ {\mathcal {C}}^2 }{(4 {\mathcal {C}}^2 + 1)({\mathcal {C}}^2 + 1)}F_{\tau }(x) \ge \frac{1}{10 {\mathcal {C}}^2} F_{\tau }(x)\ge \zeta \, , \end{aligned}$$

a contradiction to the assumption that *x* is a $\zeta$-approximate minimizer of $F_{\tau }(x)$. $\square$

We are now ready to prove Lemma 8.3.

#### Proof of Lemma 8.3

By Lemma 8.7, as $F_{\tau }(x) \ge \overline{{\mathcal {C}}}^2 \zeta \ge 10 {\mathcal {C}}^2 \zeta$,

$$\begin{aligned} \alpha \ge \frac{\sqrt{F_{\tau }(x)}}{2 {\mathcal {C}}} \ge \frac{\overline{{\mathcal {C}}}\sqrt{\zeta }}{2{\mathcal {C}}} \cdot \ge 32 n \cdot \kappa ({\mathcal {X}}_{A}) \cdot \sqrt{\zeta }. \end{aligned}$$

Thus,

$$\begin{aligned} \varepsilon \alpha = \frac{ \alpha }{ 8n\cdot \kappa ({\mathcal {X}}_{A})} \ge 4 \sqrt{\zeta }. \end{aligned}$$

Lemma 8.6 now gives $\theta (x, \pi , \sigma ) \le n\sqrt{ \zeta } / 2$, proving (i).

In addition, the definition of $F_{\tau }(x)$ implies that $\Vert Ax - b\Vert _2^2 \le 2 \Vert A\Vert _1^2 \cdot F_{\tau }(x)$. The assumption $F_{\tau }(x) \le 2 \overline{{\mathcal {C}}}^2 \zeta$ implies that

$$\begin{aligned} \Vert Ax - b\Vert _1 \le \sqrt{m} \cdot \Vert Ax - b\Vert _2 \le \sqrt{2m} \Vert A\Vert _1 \cdot \sqrt{F_{\tau }(x)} \le 2 \Vert A\Vert _1\cdot \overline{{\mathcal {C}}}\sqrt{m \zeta }\, , \end{aligned}$$

proving (ii).

Finally,

$$\begin{aligned} \Vert \pi \Vert _{\infty }&= \frac{\Vert c\Vert _{\infty }}{\alpha \Vert A\Vert _1^2}\left\| Ax - b\right\| _{\infty } \le \frac{2{\mathcal {C}}\Vert c\Vert _{\infty } }{\overline{{\mathcal {C}}}\sqrt{\zeta }\Vert A\Vert _1^2} \Vert Ax - b\Vert _{2} \\&\le 4 {\mathcal {C}}\Vert c\Vert _{\infty } / \Vert A\Vert _1 = 4n \sqrt{m} \cdot \kappa ({\mathcal {X}}_{A})\cdot \Vert c\Vert _{\infty }\, , \end{aligned}$$

proving (iii). $$\square $$

### Convergence speed of R-FGM

Let us define $B\in {\mathbb {R}}^{(m+1)\times (n+1)}$, ${\tilde{u}},{\tilde{b}}\in {\mathbb {R}}^{m+1}$ as

$$\begin{aligned} B{:}{=}\begin{pmatrix} A&  0 \\ \Vert A\Vert _1 {\hat{c}}^\top &  \Vert A\Vert _1 \end{pmatrix}\, ,\quad {\tilde{u}}{:}{=}\begin{pmatrix}u\\ M \end{pmatrix}\, \quad {\tilde{b}}{:}{=}\begin{pmatrix}b \\ \Vert A_1\Vert \tau \end{pmatrix} \end{aligned}$$

for sufficiently large *M*. With this notation, minimizing $F_{\tau }(x)$ over $0\le x\le u$ can be written in the form

23 $$\begin{aligned} \begin{aligned} \min \frac{1}{2\Vert A\Vert _1^2} \cdot \left\| B\begin{pmatrix}x\\ t\end{pmatrix}-{\tilde{b}}\right\| _2^2\\ 0\le \begin{pmatrix}x\\ t\end{pmatrix}\le {\tilde{u}}\, . \end{aligned} \end{aligned}$$

We let ${\tilde{F}}_{\tau }(x,t)$ denote the objective function. We restate Lemma 8.1 for convenience.

#### Lemma 8.1

After $O\left( k \sqrt{n} m^2 \Vert A\Vert _1^2 \cdot {\bar{\kappa }}^2({\mathcal {X}}_{A}) / \varepsilon \right)$ iterations, R-FGM returns an $\textrm{e}^{-2k} \Vert b \Vert _2^2 / (2 \Vert A\Vert _1^2)$-approximate minimizer of $F_{\tau }$.

We use the starting point $(x^0, t^0) = (0, \tau )$. The bound follows from Theorem 2.5, with the smoothness and quadratic growth bounds as below.

#### Lemma 8.9

The function ${\tilde{F}}_{\tau }(x,t)$ is $(2n+2)$-smooth.

#### Proof

We can bound the smoothness parameter as $\Vert B\Vert _2^2 / \Vert A\Vert _1^2\le (n + 1)\Vert B\Vert _1^2 / \Vert A\Vert _1^2 \le (n +1) (\max _i (A_i + \Vert A\Vert _1{\hat{c}}_i ))^2 / \Vert A\Vert _1^2 \le 2(n +1)$. $\square$

#### Lemma 8.10

(Quadratic growth)

$$\begin{aligned} \kappa ({\mathcal {X}}_{B}) \le 2(m+1)\Vert A\Vert _1 \cdot {\bar{\kappa }}^2({\mathcal {X}}_{A}) \cdot \frac{1}{\varepsilon }\, . \end{aligned}$$

Consequently, the function ${\tilde{F}}_{\tau }(x,t)$ has $\varepsilon ^2 \big / \big [64 m^4 \cdot \Vert A\Vert _1^4\cdot {\bar{\kappa }}^4({\mathcal {X}}_{A})\big ]$-quadratic growth.

The proof is based on the following lemma.

#### Lemma 8.11

For a matrix $A\in {\mathbb {Q}}^{m\times n}$ and a vector $d\in {\mathbb {R}}^{n}$, let $$K=\begin{pmatrix}A\\ d^\top \end{pmatrix}$$. Then, every elementary vector in ${\mathcal {F}}(K)$ is either an elementary vector in ${\mathcal {F}}(A)$, or the sum of two conformal elementary vectors in ${\mathcal {F}}(A)$. Further, if $d\in {\mathbb {Z}}^n$, $d\ne {\textbf{0}}$, then

$$\begin{aligned} \kappa (K)\le 2(m+1)\cdot \Vert d\Vert _\infty \cdot {\bar{\kappa }}^2(A)\, . \end{aligned}$$

#### Proof

Let *z* be an elementary vector in ${\mathcal {F}}(K)$, and consider a generalized circuit-path decomposition of *z* w.r.t. *A*,

$$\begin{aligned} z=\sum _{k=1}^h g^k\, , \end{aligned}$$

where $h\le n$ and $g^1,g^2,\ldots ,g^h\in {\mathcal {F}}({A})$ are elementary vectors that conform to *z*. Further, for each $i=1,2,\ldots ,h-1$, $\textrm{supp}(g^i)\setminus \cup _{j=i+1}^h \textrm{supp}(g^j)\ne \emptyset$.

The first statement follows by showing $h \le 2$. The proof is by contradiction: suppose $h > 2$, and consider $g^2$ and $g^3$.

First, we observe that $$\textrm{supp}(g^2) \cup \textrm{supp}(g^3) \subsetneq \textrm{supp}(z)$$, because $$\textrm{supp}(g^1)\setminus \cup _{j=2}^h \textrm{supp}(g^j)\ne \emptyset $$. Next, we show that $$\left\langle c,g^2\right\rangle \ne 0$$ and $$\left\langle c,g^3\right\rangle \ne 0$$. For if one of them equals 0, for example, $$\left\langle c,g^2\right\rangle = 0$$, then as $$g^2$$ is elementary, $$A g^2 = 0 $$ also, which implies *z* is not an elementary vector of $${\mathcal {F}}(K)$$, given that $$g_2$$ has a strictly smaller support than *z*.

To obtain a contradiction, we consider $g^{23} = \left\langle c,g^2\right\rangle g^3 - \left\langle c,g^3\right\rangle g^2$, which is non-zero as $\textrm{supp}(g^2)\setminus \cup _{j=3}^h \textrm{supp}(g^j)\ne \emptyset$ and $\left\langle c,g^3\right\rangle \ne 0$. $g^{23}$ also has a strictly smaller support than *z*, as $\textrm{supp}(g_2) \cup \textrm{supp}(g_3) \subsetneq \textrm{supp}(z)$. In addition, $\left\langle c,g^{23}\right\rangle = 0$ and $A g^{23} = 0$. This implies *z* is not an elementary vector of ${\mathcal {F}}(K)$, which provides a contradiction.

Let us now turn to the second statement: assume that $d\in {\mathbb {Z}}^n$. Take an elementary vector *z* in ${\mathcal {F}}(K)$ such that $\kappa (K)=|z_i/z_j|$ for some $i,j\in \textrm{supp}(z)$. If $z\in {\mathcal {F}}(A)$, then $\kappa (K)\le \kappa (A)$, and hence the bound follows.

Otherwise, *z* is the sum of two elementary vectors in ${\mathcal {F}}(A)$. After appropriately scaling *z*, it can be written in the form $z=\lambda _1 g^1+\lambda _2 g^2$ with $g^1,g^2\in {\bar{{\mathcal {F}}}}(A)$, i.e., they are integer vectors such that the largest common divisor of their entries is 1. Also, $\lambda _1,\lambda _2\ne 0$. Further, we must have $0=\left\langle d,z\right\rangle =\lambda _1\left\langle d,g^1\right\rangle +\lambda _2\left\langle d,g^2\right\rangle$. After possibly another scaling of *z*, we get $\lambda _1=\left\langle d,g^2\right\rangle$ and $\lambda _2=-\left\langle d,g^1\right\rangle$, that is,

$$\begin{aligned} z=\left\langle d,g^2\right\rangle g^1-\left\langle d,g^1\right\rangle g^2\, . \end{aligned}$$

By the definition of ${\bar{\kappa }}(A)$, $\Vert g^1\Vert _\infty , \Vert g^2\Vert _\infty \le {\bar{\kappa }}(A)$. By the integrality of $g^1$ and $g^2$,

$$\begin{aligned} 1\le |\left\langle d,g^2\right\rangle |, |\left\langle d,g^1\right\rangle |\le (m+1)\cdot \Vert d\Vert _\infty \cdot {\bar{\kappa }}(A)\, . \end{aligned}$$

Thus, all nonzero entries of *z* have $1\le |z_i|\le 2(m+1)\cdot \Vert d\Vert _\infty \cdot {\bar{\kappa }}(A)\cdot \kappa (A)$ , implying the claim since $\kappa (A)\le {\bar{\kappa }}(A)$. $\square$

#### Proof of Lemma 8.10

The bound on the quadratic growth parameter follows from the circuit imbalance bound: By Lemma 2.4, ${\tilde{F}}_{\tau }(x,t)$ has $1 / \theta _{2,2}^2(B)$-quadratic growth, and, by Lemma 3.6, $\theta _{2,2}(B) \le (m+1)\cdot \kappa ({\mathcal {X}}_{B})$.

Let us now show the circuit imbalance bound. Recall that $${\mathcal {X}}_{A} = \ker \begin{pmatrix} A&-I_m\end{pmatrix}$$. Since arbitrarily scaling the rows of a matrix does not change the kernel and thus does not affect the circuit imbalances, we can write $${\mathcal {X}}_{B} =\ker \begin{pmatrix}B&-I_m\end{pmatrix}=\ker (H)$$ for

$$\begin{aligned}H:==\begin{pmatrix} A & \quad 0& \quad -I_m& \quad 0 \\ \frac{1}{\varepsilon }{\hat{c}}^\top & \quad \frac{1}{\varepsilon } & \quad 0 & \quad - {\frac{1}{\Vert A\Vert _1\cdot \varepsilon }} \end{pmatrix}\, .\end{aligned}$$

Let $H'$ be the matrix arising from *H* by deleting the last column, and let us also define $$D=\begin{pmatrix} A&0&-I_m\end{pmatrix}$$.

Clearly, ${\bar{\kappa }}({\mathcal {X}}_{A}) = {\bar{\kappa }}(D)$. Recall from (9) that $1/\varepsilon$ is defined to be an integer. Hence, Lemma 8.11 is applicable to the matrix $H'$. Note that the last row of this matrix has $\ell _\infty$ norm $1/\varepsilon$, since $\Vert {\hat{c}}\Vert _\infty =1$. Therefore, $\kappa (H')\le 2(m+1)\cdot {\bar{\kappa }}({\mathcal {X}}_A) \cdot \frac{1}{\varepsilon }$.

We show that $\kappa (H)\le \Vert A\Vert _1\cdot \kappa (H')$; this implies the statement. Indeed, *H* arises from $H'$ by duplicating one of its columns and scaling it by $1/\Vert A\Vert _1$. Duplicating columns does not affect the circuit imbalance, whereas multiplying a column by any constant ${1/}\alpha$ may increase it by at most a factor $\alpha$. This completes the proof. $\square$

## Analysis of dual certificates

We now present the proof of Lemma 4.6 and Theorem 4.7 on $\delta$-certificates.

### Lemma 4.6

Let $A\in {\mathbb {R}}^{m\times n}$, $b\in {\mathbb {R}}^m$, $c,u\in {\mathbb {R}}^n$, and let $x\in [{\textbf{0}},u]$.

(i) If there is a $\delta$-certificate for *x* for some $\delta \ge 0$, then *x* is $(4n+2)\delta$-optimal.
(ii) Suppose $0\le \delta ^\textrm{feas}\cdot n\cdot \kappa ({\mathcal {X}}_A) \cdot \Vert A\Vert _1 \le \delta ^\textrm{opt}$. If *x* is a $\delta ^\textrm{feas}$-feasible and $\delta ^\textrm{opt}$-optimal solution, then there exists a $\delta ^\textrm{opt}$-certificate for *x*.

### Proof

**Part (i)**  Let $(\pi ,w^-,w^+)$ denote the $\delta$-certificate. This is a feasible solution to Dual(*A*, *b*, *c*, *u*), and therefore $\Phi (A,b,c,u)\ge \left\langle b,\pi \right\rangle -\left\langle u,w^+\right\rangle$. Thus, using the properties of $\delta$-certificates, we get the bound

$$\begin{aligned} \begin{aligned} \left\langle c,x\right\rangle -\Phi (A,b,c,u)&\le \left\langle c,x\right\rangle -\left\langle b,\pi \right\rangle +\left\langle u,w^+\right\rangle \\&=\left\langle c-A^\top \pi +w^+,x\right\rangle +\left\langle {A} x-b,\pi \right\rangle +\left\langle u-x,w^+\right\rangle \\&=\left\langle w^-,x\right\rangle +\left\langle w^+,u-x\right\rangle +\left\langle {A} x-b,\pi \right\rangle \\&\le (4n+2)\delta \Vert c\Vert _\infty \, . \end{aligned} \end{aligned}$$

***Part (ii)***

Let ${{\bar{b}}}=Ax$. By Lemma 6.3, and the assumption on $\delta ^\textrm{feas}$, it follows that

$$\begin{aligned}  &   |\Phi (A,b,c,u)-\Phi (A,{{\bar{b}}},c,u)|\le \kappa ({\mathcal {X}}_A)\cdot \Vert c\Vert _1\cdot \Vert b-{{\bar{b}}}\Vert _1\le n\\  &   \quad \cdot \kappa ({\mathcal {X}}_A)\cdot \Vert c\Vert _\infty \cdot \Vert A\Vert _1\cdot \delta ^\textrm{feas}\le \delta ^\textrm{opt}\cdot \Vert c\Vert _\infty \, , \end{aligned}$$

Thus, *x* is $2\delta ^\textrm{opt}$-optimal for $\textrm{LP}(A,{{\bar{b}}},c,u)$. Let us select an optimal dual solution $({\bar{\pi }},{{\bar{w}}}^-,{{\bar{w}}}^+)$ to $\textrm{Dual}(A,{{\bar{b}}},c,u)$. Thus, $\left\langle {{\bar{b}}},{\bar{\pi }}\right\rangle -\left\langle u,\bar{w}^+\right\rangle =\Phi (A,{{\bar{b}}},c,u)$. As in part (i), we get

$$\begin{aligned} \begin{aligned} 2\delta ^\textrm{opt}\Vert c\Vert _\infty&\ge \left\langle c,x\right\rangle -\Phi (A,{{\bar{b}}},c,u)= \left\langle c,x\right\rangle -\left\langle {{\bar{b}}},{\bar{\pi }}\right\rangle +\left\langle u,{{\bar{w}}}^+\right\rangle \\&=\left\langle {{\bar{w}}}^-,x\right\rangle +\left\langle {{\bar{w}}}^+,u-x\right\rangle +\left\langle A x-{{\bar{b}}},{\bar{\pi }}\right\rangle \, . \end{aligned} \end{aligned}$$

Since all terms here are nonnegative, we get that $({\bar{\pi }},{{\bar{w}}}^-,{{\bar{w}}}^+)$ is a feasible solution to the LP

24 $$\begin{aligned} \begin{aligned} A^\top \pi&+w^--w^+= c\,\\ 0\le w^-_i&\le \frac{2\delta ^\textrm{opt}\Vert c\Vert _\infty }{x_i}\, ,\quad \forall i\in [n]\\ 0\le w^+_i&\le \frac{2\delta ^\textrm{opt}\Vert c\Vert _\infty }{u_i-x_i}\, ,\quad \forall i\in [n]\, . \end{aligned} \end{aligned}$$

We now show that (24) has a feasible solution $(\pi ,w^-,w^+)$ with

25 $$\begin{aligned} \Vert \pi \Vert _\infty \le \kappa ({\mathcal {X}}_A)\cdot \Vert c\Vert _1\, . \end{aligned}$$

This implies that $(\pi ,w^-,w^+)$ also satisfies requirement (iii) in the definition of $\delta ^\textrm{opt}$-certificates (Definition 4.5), since

$$\begin{aligned} \kappa ({\mathcal {X}}_A)\cdot \Vert c\Vert _1\le &   \Vert c\Vert _1\cdot \delta ^\textrm{opt}/[\delta ^\textrm{feas}\cdot n\cdot \Vert A\Vert _1] \le (\Vert c\Vert _1/n) \cdot \delta ^\textrm{opt}/\Vert Ax-b\Vert _1 \\\le &   \delta ^\textrm{opt}\Vert c\Vert _\infty /\Vert Ax-b\Vert _1, \end{aligned}$$

where the first inequality uses the assumption that $\delta ^\textrm{feas}\cdot n\cdot \kappa ({\mathcal {X}}_A) \cdot \Vert A\Vert _1 \le \delta ^\textrm{opt}$, and the second inequality uses the fact that *x* is $\delta ^\textrm{feas}$.

We now show the existence of such a solution to (24). Let $c':==\max \{{\textbf{0}},c\}$ and $c'':==\max \{{\textbf{0}},-c\}$. Thus, $({\textbf{0}},c',c'')$ satisfies all inequalities in (24) except the upper bounds. Now, let $(\pi ,w^-,w^+)$ be a feasible solution to (24) such that the distance $\Vert ({\textbf{0}},c',c'')-(\pi ,w^-,w^+)\Vert _2$ is minimal.

Let ${\mathcal {Y}}{:}{=}\ker (A^\top | I_n| -I_n)$. By Lemma 3.7, and noting that duplicating columns does not affect the circuit imbalances, we see that $\kappa ({\mathcal {Y}})=\kappa ({\mathcal {X}}_A)$.

Note that $({\textbf{0}},c',c'')-(\pi ,w^-,w^+)\in {\mathcal {Y}}$. Let $\sum _{k=1}^h g^k$ be a conformal circuit decomposition of the difference of these vectors, where $g^k\in {\mathbb {R}}^{m\times n\times n}$. By the choice of $(\pi ,w^-,w^+)$, the support of each $g^k$ must contain at least one coordinate in the second block with $c'_i>\frac{2\delta ^\textrm{opt}\Vert c\Vert _\infty }{x_i}$, or in the third block with $c''_j>\frac{2\delta ^\textrm{opt}\Vert c\Vert _\infty }{u_j-x_j}$, and the corresponding component of $g^k$ must be negative.

By the conformity of the circuit decomposition, and the definition of $\kappa ({\mathcal {Y}})$, it follows that $\Vert \pi \Vert _\infty \le \kappa ({\mathcal {Y}})\cdot (\Vert c'\Vert _1+\Vert c''\Vert _1)=\kappa ({\mathcal {X}}_A)\cdot \Vert c\Vert _1$; thus, (25) holds. Therefore, $(\pi ,w^-,w^+)$ is a $\delta ^\textrm{opt}$-certificate for *x*. $\square$

### Theorem 4.7

Suppose $A\in {\mathbb {R}}^{m\times n}$, $b\in {\mathbb {R}}^m$, $x,c,u\in {\mathbb {R}}^n$, $0 \le \delta ^\textrm{feas}\cdot n\cdot \kappa ({\mathcal {X}}_A) \cdot \Vert A\Vert _1 \le \delta ^\textrm{opt}$, $\Vert A\Vert _1 \ge 1$ and $x\in [{\textbf{0}},u]$ is both $\delta ^\textrm{feas}$-feasible and $\delta ^\textrm{opt}$-optimal. Then there is an algorithm Dual-Certificate $(x,A,b,c,u,\delta ^\textrm{feas},\delta ^\textrm{opt})$ which on such inputs finds a $2\cdot \delta ^\textrm{opt}$-certificate for *x* in $O\big (m \sqrt{n} \cdot \Vert A\Vert _1 \cdot \kappa ({\mathcal {X}}_A)\cdot \log (n \Vert c\Vert _1 / \delta ^\textrm{opt})\big )$ iterations of R-FGM.

### Proof

Similarly to the feasibility algorithm in Theorem 4.1, we use R-FGM on a convex quadratic minimization problem. Namely, we consider the problem

$$\begin{aligned} \begin{aligned} \min&\frac{1}{2}\left\| A^\top \pi +w^--w^+-c\right\| ^2\, \\ 0&\le w^-_i\le \frac{2\delta ^\textrm{opt}\Vert c\Vert _\infty }{x_i}\, ,\quad \forall i\in [n]\, ,\\ 0&\le w^+_i\le \frac{2\delta ^\textrm{opt}\Vert c\Vert _\infty }{u_i-x_i}\, ,\quad \forall i\in [n]\, ,\\ -\frac{2\delta ^\textrm{opt}\Vert c\Vert _\infty }{\Vert Ax-b\Vert _1}&\le \pi _i\le \frac{2\delta ^\textrm{opt}\Vert c\Vert _\infty }{\Vert Ax-b\Vert _1}\, , \quad \forall i\in [n]\, . \end{aligned} \end{aligned}$$

Lemma 4.6(ii) guarantees the existence of a $\delta ^\textrm{opt}$-certificate, and using Definition 4.5(i), we can deduce that the optimal value for this program is 0.

We run R-FGM, starting from the all zero solution to find an $\varepsilon$-approximate solution, where $\varepsilon {:}{=}\frac{1}{2} \cdot \left( \frac{2\delta ^\textrm{opt}\Vert c\Vert _\infty }{\Vert u\Vert _1}\right) ^2$. Note that $\mu :==\sqrt{2\varepsilon }$ is smaller than any of the bounds in the box constraints above. Hence, we can modify the solution to $( \pi , {\tilde{w}}^-,{\tilde{w}}^+)$ such that $A^\top \pi +{\tilde{w}}^--{\tilde{w}}^+=c$, and this solution violates the box constraints by at most a factor 2. Thus, $(\pi , {\tilde{w}}^-,\tilde{w}^+)$ is a $2\delta ^\textrm{opt}$-certificate.

By Lemma 2.4, the quadratic growth parameter is $1 / \theta _{2,2}^2(A^\top |I_n|-I_n)$, which is larger than $1 / m^2 \kappa ^2({\mathcal {X}}_A)$. This bound follows by Lemma 3.7 and the fact that duplicating columns does not affect the circuit imbalances. In addition, by Lemma 2.2, the smoothness parameter is $(\Vert A\Vert _2 + 1)^2$ as

$$\begin{aligned} \Vert (A^\top |I_n|-I_n)\Vert _2&= \max _{p, q, r} \frac{\sqrt{\Vert A^\top p\Vert _2^2 + \Vert q\Vert _2^2 + \Vert r\Vert _2^2}}{\sqrt{\Vert p\Vert _2^2 + \Vert q\Vert _2^2 + \Vert r\Vert _2^2}}\\  &\le \max _{p, q, r} \frac{\sqrt{\Vert A^\top p\Vert _2^2}}{\sqrt{\Vert p\Vert _2^2 }} + \frac{\sqrt{ \Vert q\Vert _2^2 + \Vert r\Vert _2^2}}{\sqrt{ \Vert q\Vert _2^2 + \Vert r\Vert _2^2}} \le \Vert A\Vert _2 + 1. \end{aligned}$$

Given this, by Theorem 2.5, the total number of iterations of R-FGM is at most $O\big (m \sqrt{n} \Vert A\Vert _1 \cdot \kappa ({\mathcal {X}}_A)\cdot \log (n \Vert u\Vert _1 / \delta ^\textrm{opt})\big )$. $\square$

## Hoffman constant example for the self-dual embedding

We show that for the self-dual embedding (2), the Hoffman constant of the corresponding matrix can be unbounded.

### Lemma 10.1

Let $A\in {\mathbb {R}}^{m\times n}$, $b\in {\mathbb {R}}^m$, $u\in {\mathbb {R}}^n$ with ${\mathcal {P}}_{A,b,u}\ne \emptyset$ being at least 2-dimensional. Then, for any $M>0$, there exist a cost function $c\in {\mathbb {R}}^n$ such that the Hoffman constant is $\theta _{2,2}({\mathcal {S}}_c)\ge M$ for the system

$$\begin{aligned} {\mathcal {S}}_c = \left\{ (x, \pi , w^-, w^+) ~\Big |\, \, ~ \right.&x \in {\mathcal {P}}_{A,b,u} \, ; A^\top \pi + w^--w^+=c\, ;\\  &\left. \left\langle c,x\right\rangle -\left\langle b,\pi \right\rangle +\left\langle u,w^+\right\rangle =0\, ; w^-,w^+\ge 0\,\right\} \, . \end{aligned}$$

### Proof

Let us denote ${\mathcal {P}}={\mathcal {P}}_{A,b,u}$, and consider any facet ${\mathcal {F}}$ of ${\mathcal {P}}_{A,b}$ corresponding to the linear equation $\left\langle r,x\right\rangle = v$ for some $r \in {\mathbb {R}}^n$ and $v \in {\mathbb {R}}$. We define $c\approx r$ as a perturbation of *r* with $\Vert c-r\Vert _2\le 1/M$, such that $\max \left\langle c,x\right\rangle ~\text {s.t.}~ x \in {\mathcal {P}}$ has a unique optimal solution ${{\bar{x}}}\in {\mathcal {F}}$. Let $x'\in {\mathcal {F}}\setminus \{{{\bar{x}}}\}$ be another extreme point. Let $(\pi , w^+, w^-)$ be an optimal solution to the dual of $\max \left\langle c,x\right\rangle ~\text {s.t.}~ x \in {\mathcal {P}}$. Then, for the primal-dual pair $(x', \pi , w^+, w^-)$, we have

1. $$x'\in {\mathcal {P}}$$ ;
2. $(\pi , w^+, w^-)$ is feasible: $A^\top \pi + w^--w^+=c$ and $w^+, w^- \ge 0$;
3. the optimality gap is tiny but strictly positive, i.e., $0<\left\langle c,x'\right\rangle -\left\langle b,\pi \right\rangle +\left\langle u,w^+\right\rangle \le \left\langle c,x' - {\bar{x}}\right\rangle \le \Vert c-r\Vert _2\cdot \Vert x' - {\bar{x}}\Vert _2\le \frac{1}{M} \Vert x' - {\bar{x}}\Vert _2$;
4. the distance to the nearest point in ${\mathcal {S}}_c$ is $\Vert x' - {\bar{x}}\Vert _2$.

In this case, the Hoffman constant $\theta _{2,2}({\mathcal {S}}_c)$ is at least the distance to the nearest point in ${\mathcal {S}}_c$ divided by the optimality gap, which is at least *M*. $\square$

## Footnotes

1 Different papers may use different norms and normalizations in their accuracy requirement, but these can be easily translated to each other. Such a change of the norms might incur polynomial factors, but these do not impact the asymptotic running time due to linear convergence.

2 We could also scale *A* and *b* such that $\Vert A\Vert _1=1$. However, we do not make this assumption since multiplying *A* by a constant may change ${\bar{\kappa }}({\mathcal {X}}_A)$.

3 However, the same paper shows that standard projected gradient also converges linearly albeit at a slower rate, and does not require knowing this constant.

4 Let $M\in {\mathbb {R}}^{m\times n}$ be a matroid such that $W=\ker (M)$. One can associate a linear matroid with *M* (and *W*), where a subset of [*n*] is independent if the corresponding column vectors are linearly independent. Circuits defined as above are precisely the circuits, i.e., minimal non-independent sets in this matroid.
